# Dramatic and concerted conformational changes enable rhodocetin to block α2β1 integrin selectively

**DOI:** 10.1371/journal.pbio.2001492

**Published:** 2017-07-13

**Authors:** Johannes A. Eble, Matthew McDougall, George L. Orriss, Stephan Niland, Benjamin Johanningmeier, Gottfried Pohlentz, Markus Meier, Simone Karrasch, Maria Inacia Estevão-Costa, Augusto Martins Lima, Jörg Stetefeld

**Affiliations:** 1 Institute of Physiological Chemistry and Pathobiochemistry, University of Münster, Münster, Germany; 2 Departments of Chemistry & Microbiology, University of Manitoba, Winnipeg, Manitoba, Canada; 3 Institute of Hygiene, University of Münster, Münster, Germany; Weill Cornell Medical College, United States of America

## Abstract

The collagen binding integrin α2β1 plays a crucial role in hemostasis, fibrosis, and cancer progression amongst others. It is specifically inhibited by rhodocetin (RC), a C-type lectin-related protein (CLRP) found in Malayan pit viper (*Calloselasma rhodostoma*) venom. The structure of RC alone reveals a heterotetramer arranged as an αβ and γδ subunit in a cruciform shape. RC specifically binds to the collagen binding A-domain of the integrin α2 subunit, thereby blocking collagen-induced platelet aggregation. However, until now, the molecular basis for this interaction has remained unclear. Here, we present the molecular structure of the RCγδ-α2A complex solved to 3.0 Å resolution. Our findings show that RC undergoes a dramatic structural reorganization upon binding to α2β1 integrin. Besides the release of the nonbinding RCαβ tandem, the RCγ subunit interacts with loop 2 of the α2A domain as result of a dramatic conformational change. The RCδ subunit contacts the integrin α2A domain in the “closed” conformation through its helix C. Combined with epitope-mapped antibodies, conformationally locked α2A domain mutants, point mutations within the α2A loop 2, and chemical modifications of the purified toxin protein, this molecular structure of RCγδ-α2A complex explains the inhibitory mechanism and specificity of RC for α2β1 integrin.

## Author summary

In animals, collagen-mediated platelet aggregation is an essential component of the blood’s clotting response following vascular injury. A small group of snake venom toxins belonging to the C-type lectin protein family exert their harmful effects by directly targeting this pathway. Rhodocetin (RC) is a heterotetrameric protein found in the venom of the Malayan pit viper (*C*. *rhodostoma*). RC specifically binds α2β1 integrin, the key protein required for collagen-mediated platelet aggregation. In this study, we describe the interaction between RC and α2β1 integrin at atomic resolution. This study reveals that RC undergoes a massive structural reorganization upon α2β1 integrin binding, such that RC’s αβ subunit is released from its γδ subunit and a γδ-α2β1 integrin complex is formed. The inhibitory nature of this complex can be readily explained as RC binding along the top surface of the α2β1 integrin and directly above the collagen binding site. As a result, access of collagen to its binding site is blocked, thereby preventing collagen-mediated platelet aggregation.

## Introduction

Most cellular processes depend on the formation of interactions between cells and the extracellular matrix (ECM). Key facilitators of these interactions are the integrins. They consist of 2 subunits, α and β, each of which has multiple isoforms [1,2]. The different subunit composition between integrins determines their ligand-binding specificity and functionality. Integrins are cell adhesion molecules, which are involved in a broad range of cell functions, such as proliferation, differentiation, adhesion, and migration. Defect or dysfunction of integrins, in particular of α2β1 integrin, a prominent collagen binding receptor of many cell types [3] and the only collagen binding integrin on platelets [4], may affect vascular development and angiogenesis [5], epithelial cell differentiation [6], wound repair and fibrosis [7], inflammation [8,9], and cancer and cancer therapy [10], as well as collagen-induced platelet activation, hemostasis, and thrombosis [4,11]. Therefore, α2β1 integrin has become a prominent target in drug research [12–14].

The collagen binding site is located within the α2A domain of α2β1 integrin, which is homologous to the A-domain of von Willebrand factor (vWF). The α2A domain contains a metal ion that is required for collagen binding as it is part of the binding site for the collagen triple helix [15]. In order to bind to collagen, the α2A domain undergoes a series of concerted conformational changes. In short, helix C unwinds, the N-termini of helices 6 and 7 simultaneously turn away from each other, and, finally, helix 7 moves downward against helix 1 to give the collagen binding “open” conformation, which contrasts with the previous “closed” conformation [15,16]. This likely general mechanism of molecular movement of integrin A-domains was subsequently confirmed by introducing a disulfide bridge into the A-domain of the integrin αL subunit such that this interconversion was blocked with the protein locked in either the “open” or “closed” state [17].

Integrin function can be blocked by two major classes of snake venom proteins, the disintegrins [18,19] and the C-type lectin-related proteins (CLRPs) [20,21]. In contrast to the disintegrins, which can target multiple integrins, CLRPs specifically inhibit α2β1 integrin activity [21]. The high selectivity and affinity of these snake venom proteins for α2β1 integrin make them ideal lead compounds for drug development [22–24]. Current members of the CLRP family include the proteins rhodocetin (RC), EMS16, vixapatin, sochicetin-B, lebecetin, flavocetin, and rhinocetin [25–31]. As more CLRP structures become available, it is clear that, although the supramolecular structure can vary from the basic heterodimer of EMS16 [27] to the ring-like (αβ)_4_ structures of flavocetin and convulxin [32,33], the underlying basic unit is a heterodimer consisting of 2 subunits, usually named α and β, which dimerize via their characteristic index finger loops [20,34]. Interestingly, in the case of the RC heterotetramer (αβγδ) structure [26], the αβ and γδ subunits form 2 heterodimeric pairs that are oriented orthogonally towards each other in a cruciform shape. Despite these differences, the subunits of CLRP family members are highly homologous with each other. Evolutionarily, the CLRP fold has developed from a carbohydrate recognizing domain (CRD) into a structure that specifically targets clotting factors IX and X, α2β1 integrin, and other platelet adhesion receptors [20,34–36]. Among the latter, the vWF receptor and the 2 collagen binding receptors, glycoprotein GPIV and α2β1 integrin, are targets for snake venom CLRPs, thereby inhibiting or activating platelet activation and aggregation [37,38]. Consequently, these snake venom proteins severely interfere with hemostasis [36,39]. However, the nature of the molecular mechanism by which CLRPs inhibit α2β1 integrin and by which CLRPs implement specificity towards α2β1 integrin has remained undetermined.

RC is a CLRP of the Malayan pit viper *C*. *rhodostoma* [26], and together with EMS16 from *Echis multisquamatus*, they are the only known CLRP family members proven to target the α2A domain for which atomic resolution structures are available [27,40]. Unlike the α2β1 integrin–collagen interaction, which is metal ion-dependent, the binding of RC to α2β1 integrin does not require a metal ion, which implies a different mechanism of action. In a previous study, we demonstrated that the RCαβγδ heterotetramer binds to α2β1 integrin before releasing the αβ subunit (RCαβ) from the complex [40]. In the current work, we present the molecular structure of this RCγδ-α2A domain complex and unravel the molecular mechanism of this interaction. The RC binding site overlaps with that of collagen, including the key metal ion site, thereby sterically blocking collagen binding. Moreover, a comparison with the previously determined RC structure [26] reveals that, in addition to the release of the RCαβ subunit, the RCγδ subunit undergoes a major conformational change upon integrin binding, which causes it to snap into a bent conformation like a mouse trap. In this final state, RCγδ holds the α2A domain in the “closed” conformation, allosterically unable to bind to collagen. The result is a highly efficient inhibition of α2β1 integrin-mediated attachment and signaling in cells and platelets.

## Results

### Purification and characterization of the RCγδ-α2A complex

**Fig 1:**
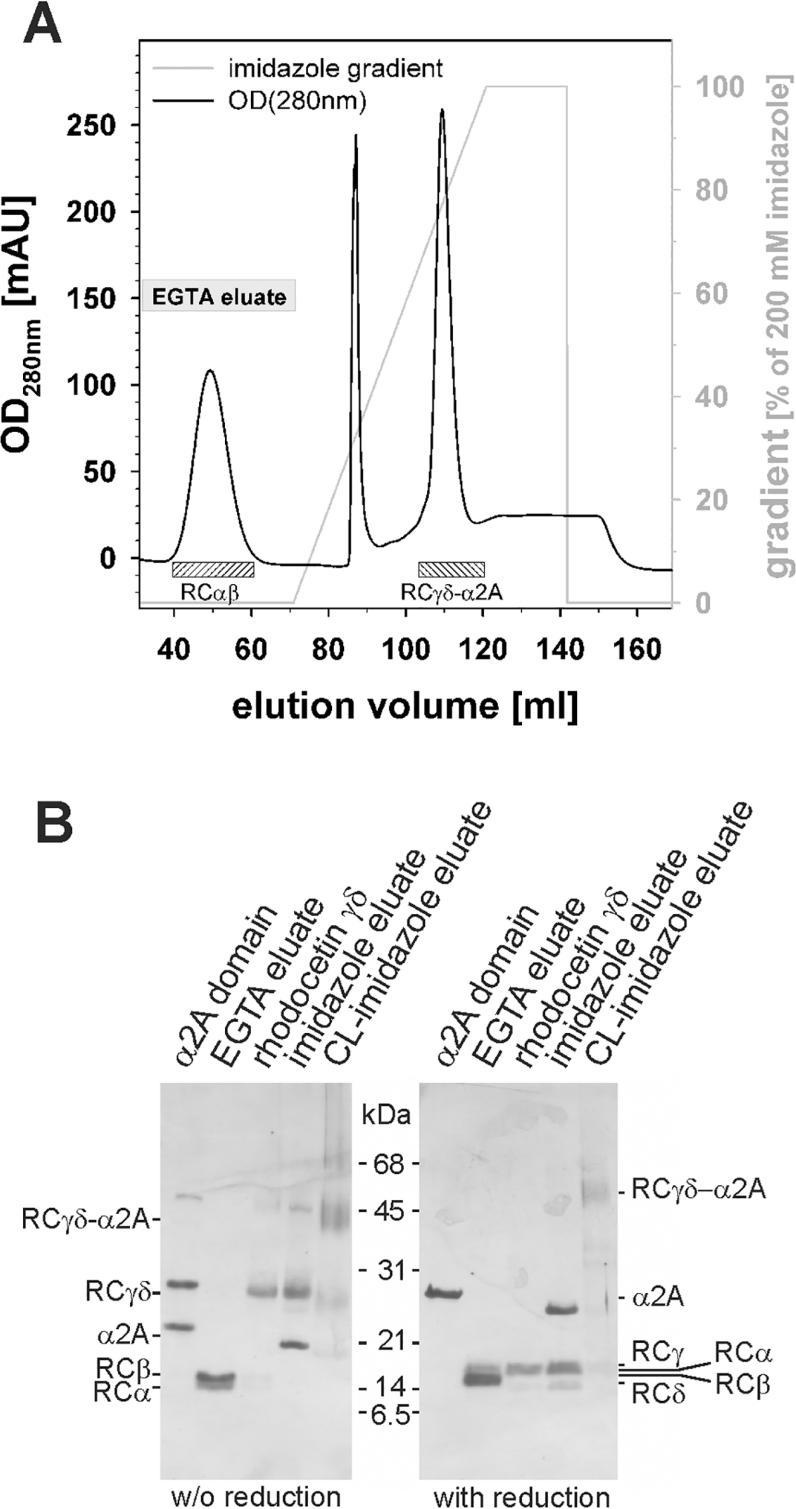
Isolation of the rhodocetin γδ-α2A complex on Ni Sepharose column. **(A)** Elution profile of the Ni Sepharose affinity chromatography column. The RCγδ-α2A complex was formed on a Ni Sepharose column by subsequently loading the oligo His-tagged α2A domain and RCαβγδ. RCαβ and the RCγδ-α2A complex were eluted with EGTA and an imidazole gradient, respectively. (**B**) SDS-PAGE of eluate fractions (lanes “EGTA eluate” and “imidazole eluate”), in comparison to isolated control proteins (lanes “α2A domain” and “rhodocetin γδ”), under nonreducing and reducing conditions and stained with silver. Note that the trypsin-trimmed RCγδ-α2A complex showed a slightly reduced size of the α2A domain due to the proteolytic removal of the His_6_-tag. The physical contact of co-eluted rhodocetin (RC) γδ and α2A domain was analytically proven by cross-linkage with 0.5 mM BS^3^ (lane “CL-imidazole eluate”).

To isolate RC in complex with the integrin α2A domain, recombinant α2A domain was immobilized to Ni Sepharose resin via its His_6_-tag. Thereafter, an RC-rich protein fraction of *C*. *rhodostoma* venom was applied to this column, resulting in the formation of the complex of α2A with tetrameric RC (RCαβγδ) that still bound to the column. Treatment with 5 mM EGTA resulted in the dissociation of the α2A domain bound RC tetramer and the release of RCαβ from the complex, which was eluted from the column. In contrast, RCγδ remained firmly attached to the column bound α2A (Fig 1). This RCγδ-α2A complex was then eluted with a linear gradient of imidazole (Fig 1A). Its His_6_-tag was cleaved by trypsinolysis, and the excess α2A was removed by size-exclusion chromatography. The close physical contact of both partners within the RCγδ-α2A complex was proven by cross-linkage with 0.5 mM bis(sulfosuccinimidyl)suberate (BS^3^) (Fig 1B).

### Molecular structure of the rhodocetin γδ-α2A complex

**Fig 2:**
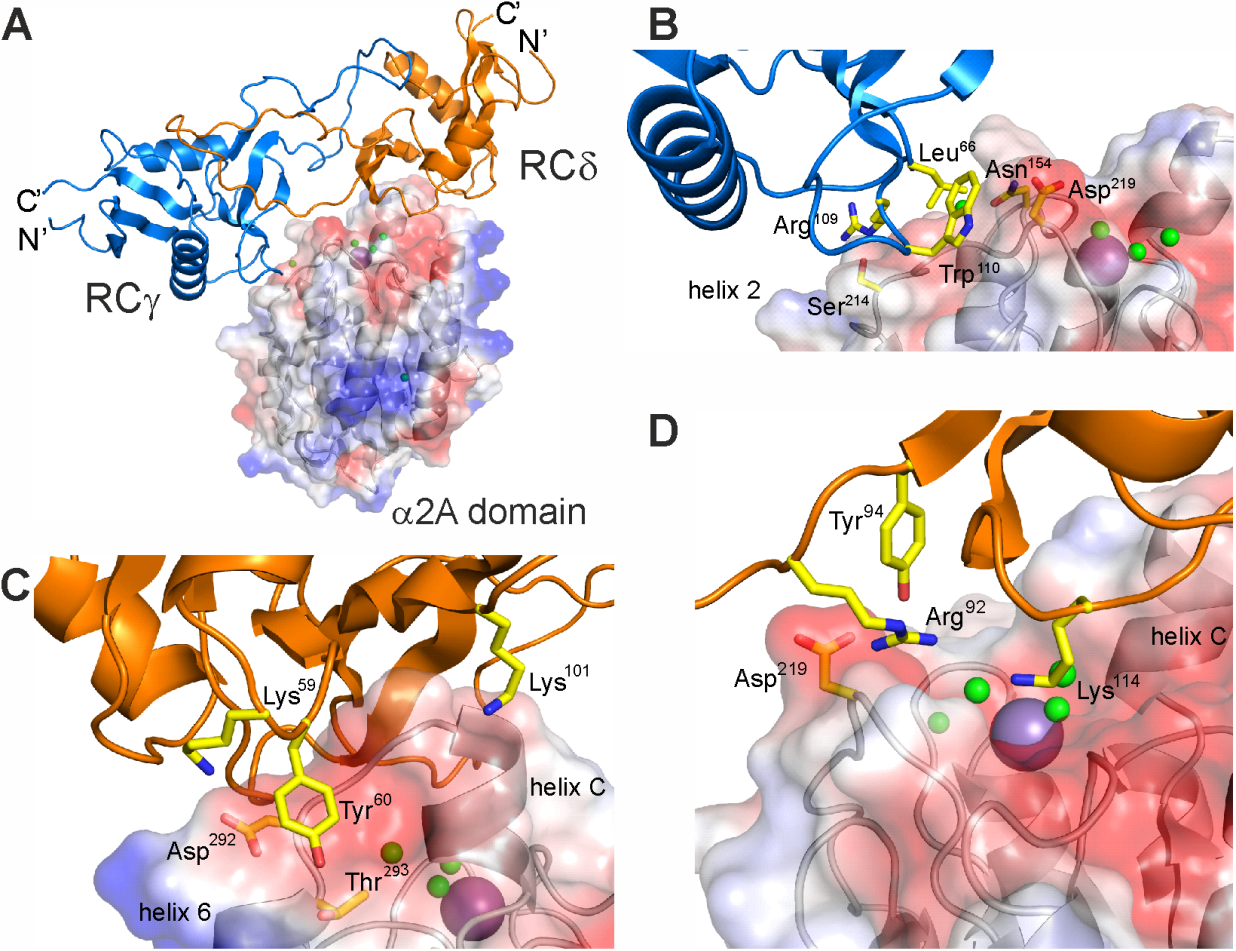
The molecular structure of the RCγδ-α2A complex. (**A**) Molecular structure of the RCγδ-α2A complex reveals that RCγδ binds on the top and lateral faces of the α2A domain. The RCγδ subunit covers the collagen binding crevice of the α2A domain, with its long axis perpendicular to the collagen–ligand interaction. **(B)** Detailed view of the interaction site between the RCγ chain and loop 2 of α2A. (**C**, **D**) Two different views of the interaction site between the RCδ subunit and helix C of α2A. The α2A domain is shown as a transparent surface in (**A**) through (**D**), with the key binding residues labelled, while the water molecules and magnesium ion are represented as green and purple spheres, respectively.

The crystal structure of the RCγδ-α2A complex was determined at 3.0 Å resolution by molecular replacement using the previously determined RCαβγδ structure (pdb:3GPR) as a search template (Fig 2). The RCγδ-α2A structure clearly showed that the RCγδ subunit bound to the top of the α2A domain directly above the metal ion-binding site, thereby sterically blocking access of collagen (Fig 2A). Both chains of RCγδ are typical CLRP folds, characterized by a globular core domain interlinked mutually by extended index finger loops. The A-domain of α2β1 integrin assumed the “closed” conformation with its central β-sheet flanked by the α-helices 3, 1, and 7 and 4, 5, and 6 on either side. The crystal structures contain 6 RCγδ-α2A complexes per asymmetric unit (S1 Fig).

We determined the total interaction surface between RCγδ and α2A in the complex to be 965 Å^2^. There were 2 interface areas on the surface of RCγδ in contact with α2A (Fig 2B–2D). First, the larger interaction site (715 Å^2^) consisted of 2 adjacent patches of 3 residues each on the RCδ subunit, K59-Y60-K101 (Fig 2C), and R92-Y94-K114 (Fig 2D), which were largely hydrophilic. Second, a smaller hydrophobic site (280 Å^2^) on the RCγ subunit consisted of the triad L66-R109-W110 that interacted with helix 3, helix 4, and loop 2 of α2A (Fig 2B).

Two complementary contact surfaces on the α2A domain extended down from helix C and the metal ion-binding site (top face) to the loop 2 sequence S^214^QYGGD^219^ (lateral face) to form an almost contiguous interface that interacted with the RCγδ subunit. The top face of α2A was approached by the RCδ subunit with its larger 2 patches containing interface (Fig 2C and 2D). The first patch comprised residues K59, Y60, and K101 of RCδ interacting with residues D292 and T293 together with the adjacent helix C of α2A. The side chains of K59 and Y60 were countered by complementary carboxylate and hydroxyl groups of D292 and T293 of α2A, while the amino group of K101 pointed towards the backbone carbonyl groups at the C-terminus of helix C. The second patch had the side chains of R92, Y94, and K114 of the RCδ subunit pointing into the collagen binding crevice of α2A. The long side chain of K114 of this protuberance sat at the entrance to the divalent cation binding site (Fig 2D) and was positioned 7.7 Å above the magnesium ion, whereas the positively charged guanidino group and the phenolic hydroxyl group of R92 and Y94 contacted the main chain carbonyl of D219 in loop 2 of α2A.

The second contact surface is the loop 2 sequence S^214^QYGGD^219^ at the lateral face of α2A, which interacted with the amino acid side chains of L66, R109, and W110 of the RCγ subunit (Fig 2B). For example, the aromatic indole ring of W110 contributed to a hydrophobic surface and interacted with the backbone chain of the glycine residues G217 and G218 together with the adjacent aspartate residue D219 within loop 2 of the α2A domain (Fig 2B). In addition, L66 of RCγ contacted N154 of loop 1 of the α2A domain. The final RCγ residue of the triad R109 made contact with the S214 side chain of α2A. Taken together, the hydrophobic patch of the RCγ subunit predominantly interacted with the loop 2 sequence S^214^QYGGD^219^ of α2A. This loop 2 sequence immediately preceded residue T221, which was part of the metal ion binding site of α2A. A key residue with regard to the interface between the RCγδ subunit and the α2A domain in the RCγδ-α2A complex was the loop 2 D219 of α2A, as it was part of both RC contact sites. In addition, it connected the loop 2 sequence with the collagen binding crevice and helix C of α2A. The presence of helix C in the RCγδ-α2A complex structure indicated that RC had trapped the α2A domain in the “closed” conformation, which is not capable of binding collagen [15].

### RCγδ binds the “closed” conformation of α2A

**Fig 3:**
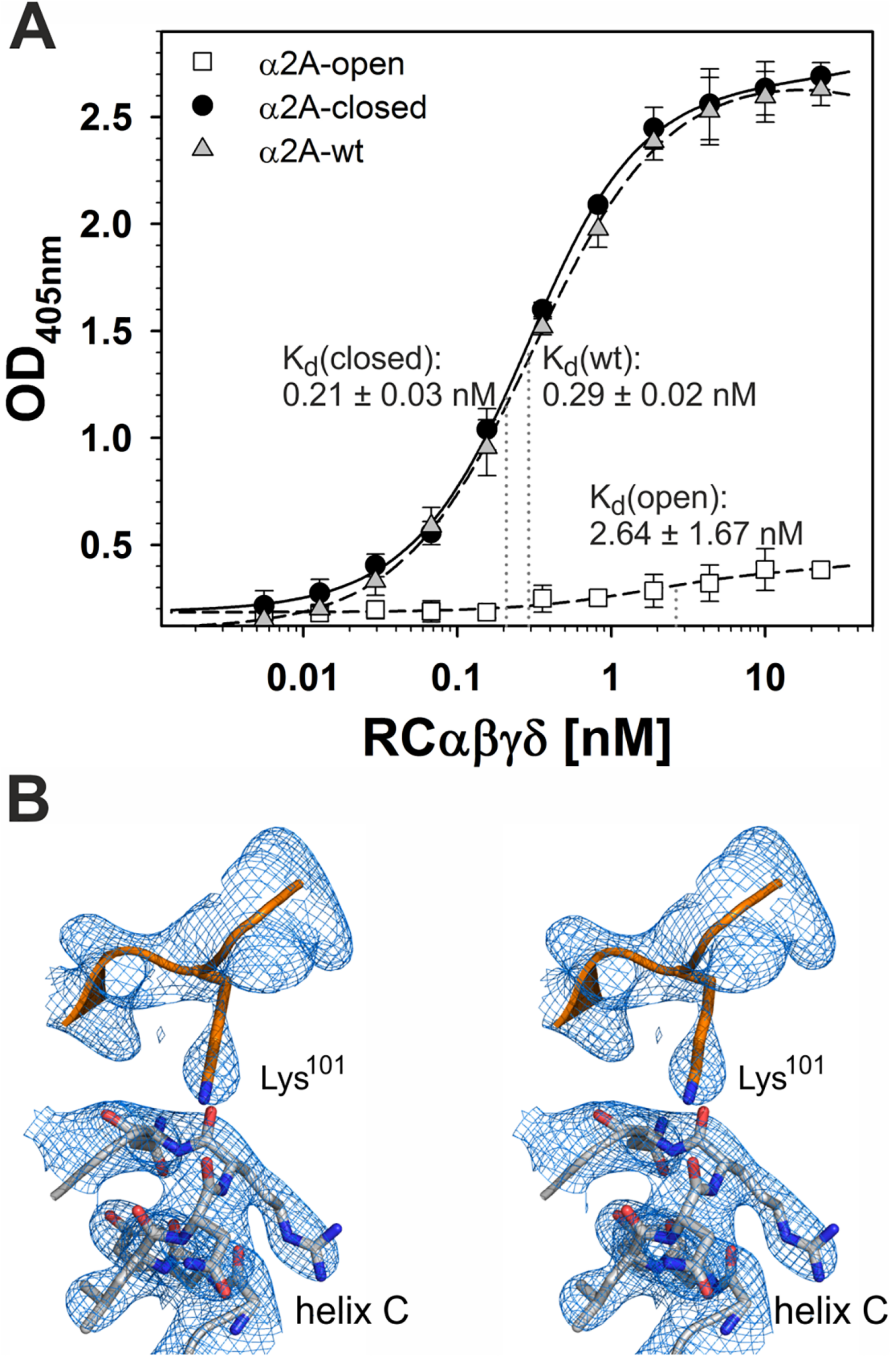
Rhodocetin (RC) recognizes the “closed” conformation but not the “open” conformation of the integrin α2A domain. **(A)** Titration of different α2A conformations with RC. The disulfide-locked conformation mutants, α2A open (□) and α2A closed (●), were immobilized to microtiter plates at 10 μg/ml. Along with immobilized α2A wild-type (wt) form (△), they were titrated with RCαβγδ. Bound RC was fixed and quantified with a rabbit RC antiserum by ELISA with a photometric signal at 405 nm. The OD_405_ values were corrected for α2A domain-free, bovine serum albumin (BSA)-blocked controls. The data presented here are taken from three independent experiments, with each measurement made in duplicate. Means ± SD (*n* = 6) are shown. The K_d_ values for RC binding to the disulfide-locked conformation mutants and the wt form of α2A are indicated at the titration curves. Both “open” and “closed” conformations have significantly different K_d_ values when compared to the one of the wild type form (* *p* < 0.05, Student *t* test). The data are summarized in S1 Data. **(B)** The crystal structure of the RCγδ-α2A complex reveals that the “closed” conformation of the α2A domain with its characteristic helix C is stabilized by the bound RCδ subunit. A stereo view of the Sigma-A weighted 2Fo-Fc map at 3.0Å resolution is shown at 1.5σ contour level.

To test whether RC exclusively binds the closed conformation of α2A, we generated 2 conformationally distinct mutants in which the A-domain was held by a disulfide bridge between K168C-E318C and K168C-A325C in the open and closed conformations, respectively (S2 Fig) [17,41]. Before introducing cysteine residues at these positions, it was necessary to replace the naturally occurring original cysteine residues at position 150 and 270 with alanines. No change in binding affinity to RC was observed for this α2A-C150A,C270A double mutant. In this cysteine-free α2A domain, K168 of α-helix 1 was replaced by a cysteine residue, with a second cysteine residue introduced into α-helix 7 at either position E318 or A325. As a consequence of the newly formed disulfide bridge, the movement of helices 1 and 7 with respect to each other that occurs when α2A shifts between the “open” and “closed” conformation was blocked. Thus, the α2A domain was held in the “open” (K168C-E318C) and “closed” (K168C-A325C) conformation, respectively. The α2A mutant with the “open” conformation hardly bound to RC (Fig 3A), while RC binding to the “closed” conformation of α2A (K_d_-value: 0.21 ± 0.03 nM) was similar to that obtained with wild-type α2A (K_d_-value: 0.29 ± 0.02 nM). Our structural findings revealed that the sidechain moiety of Lys101 is oriented towards the negatively charged dipole of helix C, stabilizing the closed conformation of the α2A domain (Fig 3B).

### The epitope of the monoclonal antibody IIIG5 is unmasked in the RCγδ-α2A complex

**Fig 4:**
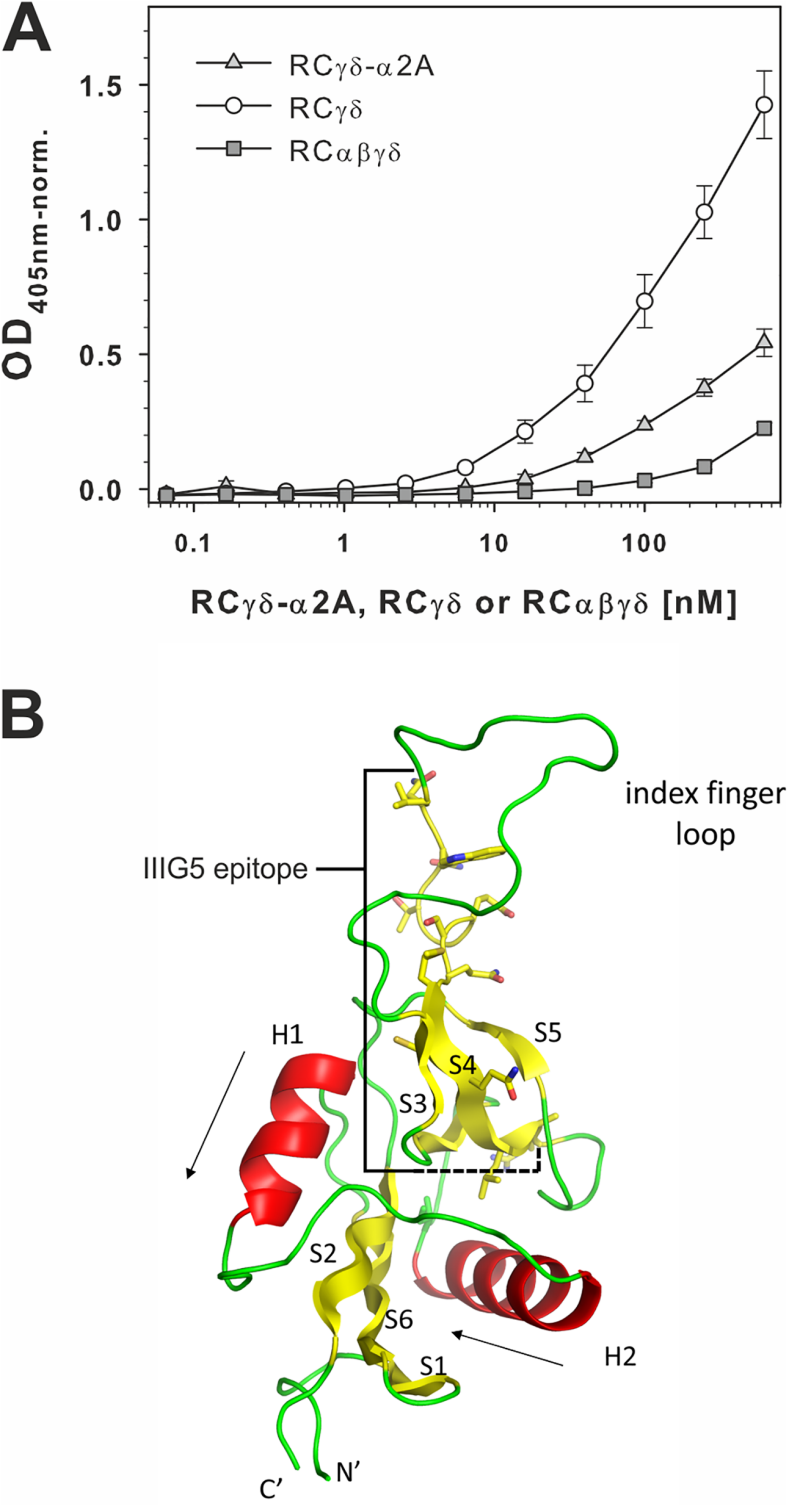
The monoclonal antibody IIIG5 recognizes its epitope within the RCγ subunit in the RCγδ-α2A complex but not in the tetrameric RCαβγδ. (**A**) The monoclonal antibody IIIG5 recognized an epitope of the RCγ subunit, which is fully accessible in the RCγδ subunit (○), partially accessible in the RCγδ-α2A complex (light gray ▲), and completely covered in the RCαβγδ tetramer (dark gray ■). IIIG5 was immobilized on microtiter plates and titrated with RCαβγδ, RCγδ-α2A complex, or RCγδ subunit. Bound rhodocetin (RC) components were fixed and detected using rabbit RC antiserum with ELISA at 405 nm. The data presented here are taken from 3 independent experiments with each measurement done in duplicate. Means ± SD are shown. The data are summarized in S1 Data. (**B**) Molecular structure of the C-type lectin-related protein (CLRP)-fold typical of all 4 RC chains. Both the γ and δ subunits of RC are very similar (Cα-RMSD 0.8Å) and feature a core structure with 2 α-helices (H1 and H2) flanked by 2 antiparallel β-sheets (S1–S2–S6 and S3–S4–S5). The amino acid residues V94–R109 of the IIIG5 epitope of RCγ are highlighted.

Among several monoclonal antibodies raised against the RCγδ subunit [40], IIIG5 belonged to the subgroup that only recognized its epitope within RCγδ after its complexation with α2A and the subsequent release of the RCαβ subunit (Fig 4A). This became evident when the antibody was immobilized and its ability to capture RCαβγδ, RCγδ- α2A, or RCγδ out from solution was probed. IIIG5 gave a binding signal with the RCγδ- α2A complex and RCγδ but not with the RC tetramer alone. Of the 2 RC species capable of binding the IIIG5 antibody, the RCγδ subunit gave the highest binding signal (Fig 4A). The most probable explanation for these results was that the IIIG5 epitope was fully accessible in RCγδ, and so, we observed what approximates the maximal binding. At the other extreme, we had no binding of RCαβγδ, as the epitope was entirely masked in the tetramer. Between these 2 extremes was the RCγδ-α2A complex, in which the epitope is sufficiently exposed for IIIG5 to bind but not to the same extent as for RCγδ due to the nature of the RCγδ-α2A interaction.

The sequence epitope of IIIG5 was isolated from a tryptic digestion of RCαβγδ by affinity chromatography on an IIIG5 column and subsequently by reversed-phase high-performance liquid chromatography (HPLC). Mass spectrometry (MS) identified the γ chain sequence 94–106 as the IIIG5 epitope (S3 Fig), which was mainly located within the index finger loop of RCγ (Fig 4B). This result can be clearly explained by comparing the native RCαβγδ structure with the newly determined RCγδ-α2A complex structure. The IIIG5 epitope was covered by the RCαβ subunit in the RCαβγδ structure and only became accessible upon formation of the RCγδ-α2A complex. Moreover, the index finger loop of the RCγ underwent a major conformational change upon formation of the RCγδ-α2A complex, leading to increased accessibility of the IIIG5 epitope.

### Conformational changes within the RCγδ-dimer after α2A binding

**Fig 5:**
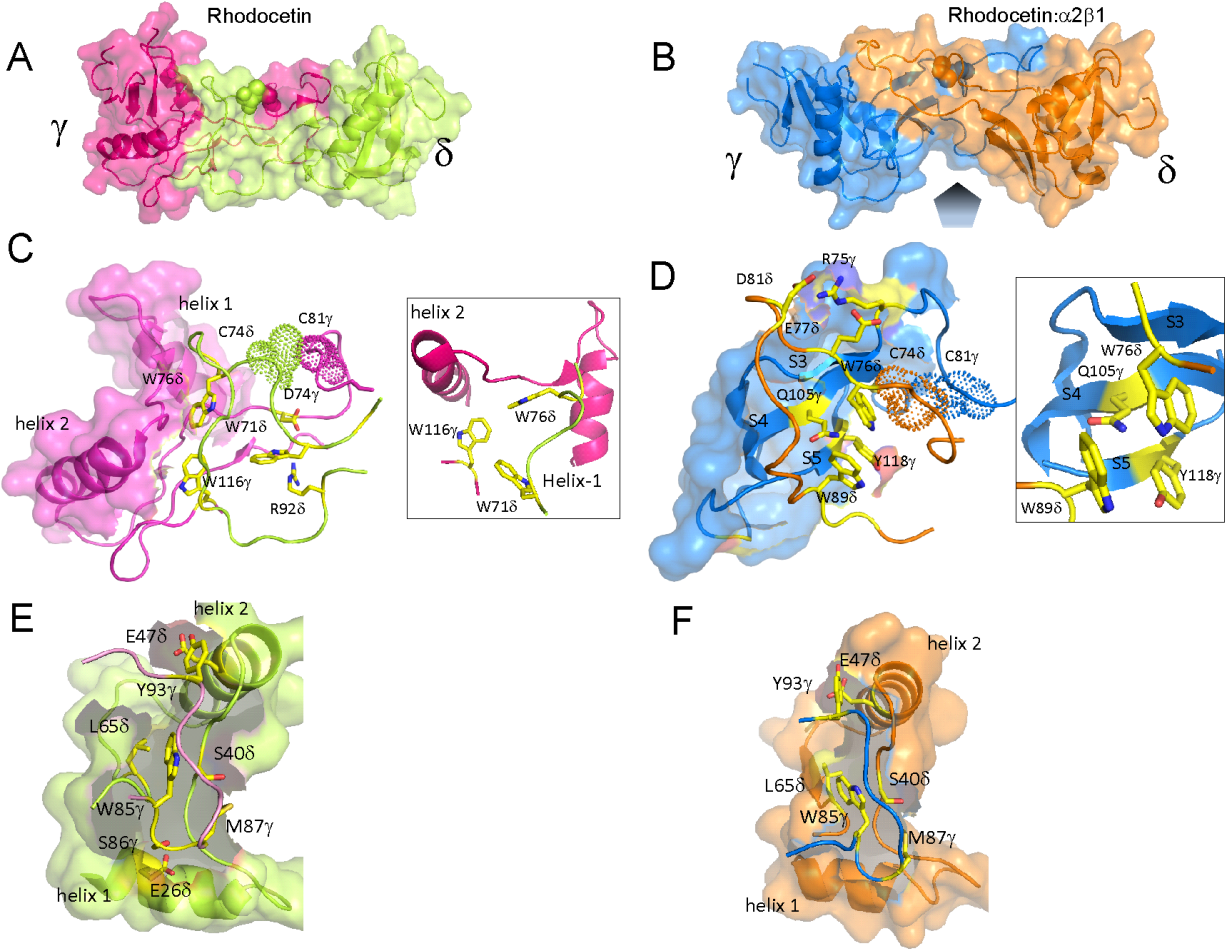
Conformational changes of RCγδ upon α2A binding. Molecular surface presentation showing the dramatic global conformational changes that occur within the γδ subunit between the RCαβγδ tetramer (**A, C, E**) and the RCγδ-α2A complex (**B, D, F**). The RC subunits γ and δ and their conformations are color-coded red (γ) and green (δ) for RCαβγδ and blue (γ) and orange (δ) for the RCγδ-α2A complex, respectively. (**A**) and (**B**) Whereas the prospective binding face towards α2A (gray pentagon approaching from the bottom in **B**) was rather flat in RCαβγδ (**A**), RC adopted a concave surface towards α2A upon formation of the RCγδ-α2A complex (**B**). In the RCαβγδ tetramer (**C**), the loop δ–core γ interface is stabilized by the 3 tryptophan residues, W116γ, W71δ, and W76δ, which form a stabilizing butterfly structure together with a salt bridge between R92δ and D74γ and a disulfide link between C81γ and C74δ, which is depicted as a dotted surface. (**D**) In RCγδ-α2A, the index finger loop from subunit δ moves towards the antiparallel sheet S3–S4–S5 of subunit γ. The disulfide bridge between C81γ and C74δ (highlighted as spheres in [**A**] through [**D**]) is unaffected, but the stabilizing butterfly is destroyed and replaced by a hydrophobic cluster of W76δ, W89δ, and Y118γ. In addition, a new salt bridge between R75γ and E77δ and D81δ is formed in place of the broken salt bridge between R92δ and D74γ. (**E, F**) A detailed view of the loop γ–core δ interaction as observed in RCαβγδ (**E**) and RCγδ-α2A complex (**F**), respectively. In both cases, the interface is highly conserved and does not alter its conformation upon the transition from RCαβγδ to RCγδ-α2A complex. The index finger loop residues of RCγ remain oriented towards the same residues of the bridge element between both helix 1 and helix 2 of the RCδ core.

The dramatic conformational changes that took place within the RCγδ subunit were readily apparent upon comparing the molecular structures of the RCγδ-α2A complex with the native RCαβγδ tetramer (Fig 5). The binding face of RCαβγδ changes from a flat surface into a concave binding surface to embrace the α2A domain (Fig 5A and 5B). This was implemented via (i) a rigid body movement of both core segments of chains γ and δ, (ii) a dramatic re-orientation of the index finger loop of the γ subunit, which harbors the IIIG5 epitope, and, consequently, (iii) local re-orientations of key binding residues in both RC subunits (Fig 5C and 5D).

The rigid body arrangement can best be described as a flipping of helices 1 and 2 between the RCγ and RCδ subunits whilst maintaining the same relative orientation of the 2 helices within their respective core domains. An additional consequence of this rigid body movement is a conformational shift of the connecting finger loop to track the motion of the opposing core domain. As a result, the 2 core domains flipped over with respect to each other and bent towards the α2A domain to form a concave binding surface such that the RCγδ residues involved in α2A binding were brought into the correct orientation for binding the α2A domain.

The apical ends of the index finger loops were in close contact with the CLRP core element of the opposite subunit, forming the 2 interfaces: loop γ–core δ and loop δ–core γ. Whereas the former hardly changed (Fig 5E and 5F), the latter showed a dramatic shift within the RCγδ-α2A complex as compared to the RCαβγδ tetramer (Fig 5C and 5D). In the loop δ–core γ interface of the RCαβγδ tetramer (Fig 5C), a tryptophan core composed of 3 residues (W76δ, W71δ, and W116γ) together with a salt bridge between R92δ and D74γ stabilized the index finger loop of the RCδ subunit and oriented it towards the RCγ subunit core sequence connecting helices 1 and 2. However, in the RCγδ-α2A complex, the salt bridge between R92δ and D74γ found in the RCαβγδ tetramer (Fig 5C) was broken. R92δ now formed a hydrogen bond to the main chain of D219 in the α2A loop 2, and a new salt bridge was observed between R75γ and E77δ and D81δ (Fig 5D). In addition, the RCδ subunit index finger loop became embedded within the antiparallel sheet S3–S4–S5 of the RCγ core such that the indole moiety of W76δ now made van der Waals contacts to Q105γ and Y118γ (see inset Fig 5C and 5D).

**Fig 6:**
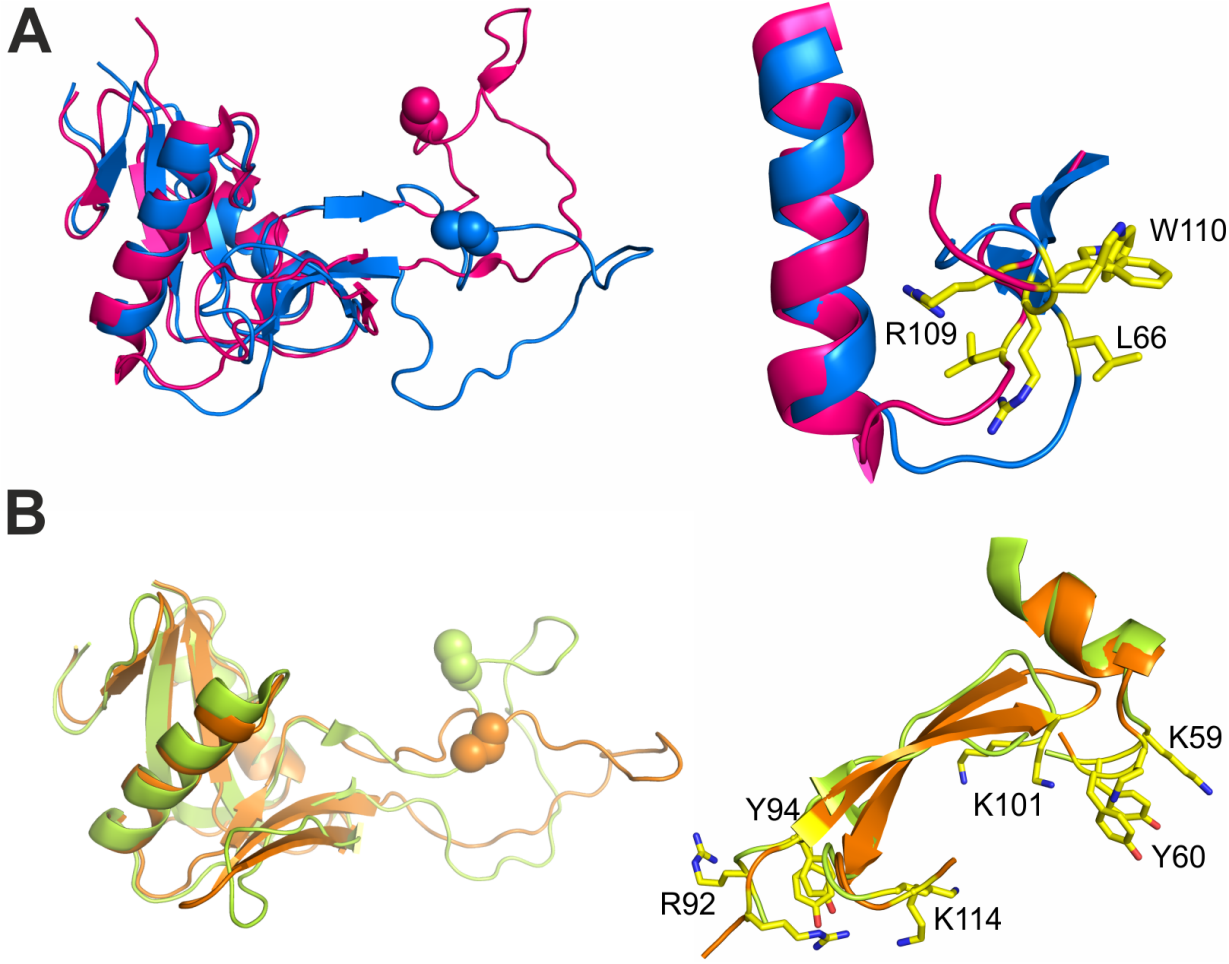
An overview of the RCγ and RCδ binding residues, depicting the local conformational changes that occur upon α2A binding. (**A**) A comparison of the RCγ subunit binding site (L66/R109/W110) between the RCαβγδ (purple) and RCγδ-α2A complex (blue) structures. Due to the global movements within the index finger swapping domain that accompany the formation of the RCγδ-α2A complex, a local repositioning of the key α2A interacting residues within RCγ takes place such that they adopt an orientation that is compatible for α2A binding. (**B**) A comparison between the 2 RCδ subunit binding sites (K59/Y60/K101 and R92/Y94/K114) between the RCαβγδ (yellow) and RCγδ-α2A complex (orange) structures. In contrast to RCγ, all the RCδ residues involved in α2A binding would be in an α2A-competent orientation in both the RCαβγδ (yellow) and RCγδ-α2A complex (orange) structures, with the exception of R92, which forms an internal salt bridge with D74γ in the RCαβγδ tetramer but interacts with D219 of α2A in the RCγδ-α2A complex.

As a result of these enormous conformational changes, especially at the loop δ–core γ interface, the rigid cores of the 2 RCγδ subunits swung towards each other by about 40°–50° around a hinge located in the center of the index finger swap domain between the cores. This global movement had 2 major consequences. First, as the RCδ subunit snapped into its new position, the 3 key residues of RCγ (L66, R109, and W110) underwent a local conformational change that transformed them into an orientation that is competent for α2A binding (Fig 6A). Second, as a consequence of the index finger loop tracking the movement of the RCγ subunit, the contact site between the RCα and RCγ subunits changed its 3D structure due to the formation of the new salt bridge between R75γ and E77δ and D81δ (Fig 5D). Consequently, the previous interface between the RCγ subunit (K^77^EQQC^81^) and the RCα subunit (N^74^KQQR^78^) became sterically blocked [26]. The movement of the RCγ subunit would also produce steric clashes with the RCβ subunit, and it is likely the combination of these 2 events that resulted in the dissociation of the RCαβ subunit from its RCγδ counterpart. In contrast, the contact site within the RCδ subunit would allow integrin binding irrespective of the conformational change of RC, as their local positions and orientations remained almost unchanged (Fig 6B). In fact, the distance between Y60δ and Y94δ within the RCδ contact sites only changed slightly, from 21.7 Å to 20.4 Å (Fig 6B), while their distances towards W110γ of the RCγ contact site were reduced from 47.5 Å to 31 Å and from 28.4 Å to 18.6 Å, respectively when comparing the structure of RCαβγδ and RCγδ-α2A complex. This illustrated how significant a reorganization of the RCγδ is required to facilitate the formation of the ultimate inhibitory RCγδ-α2A complex.

### Interaction of the RCγ subunit with loop2 of α2Adomain is essential for RC binding to the integrin

**Fig 7:**
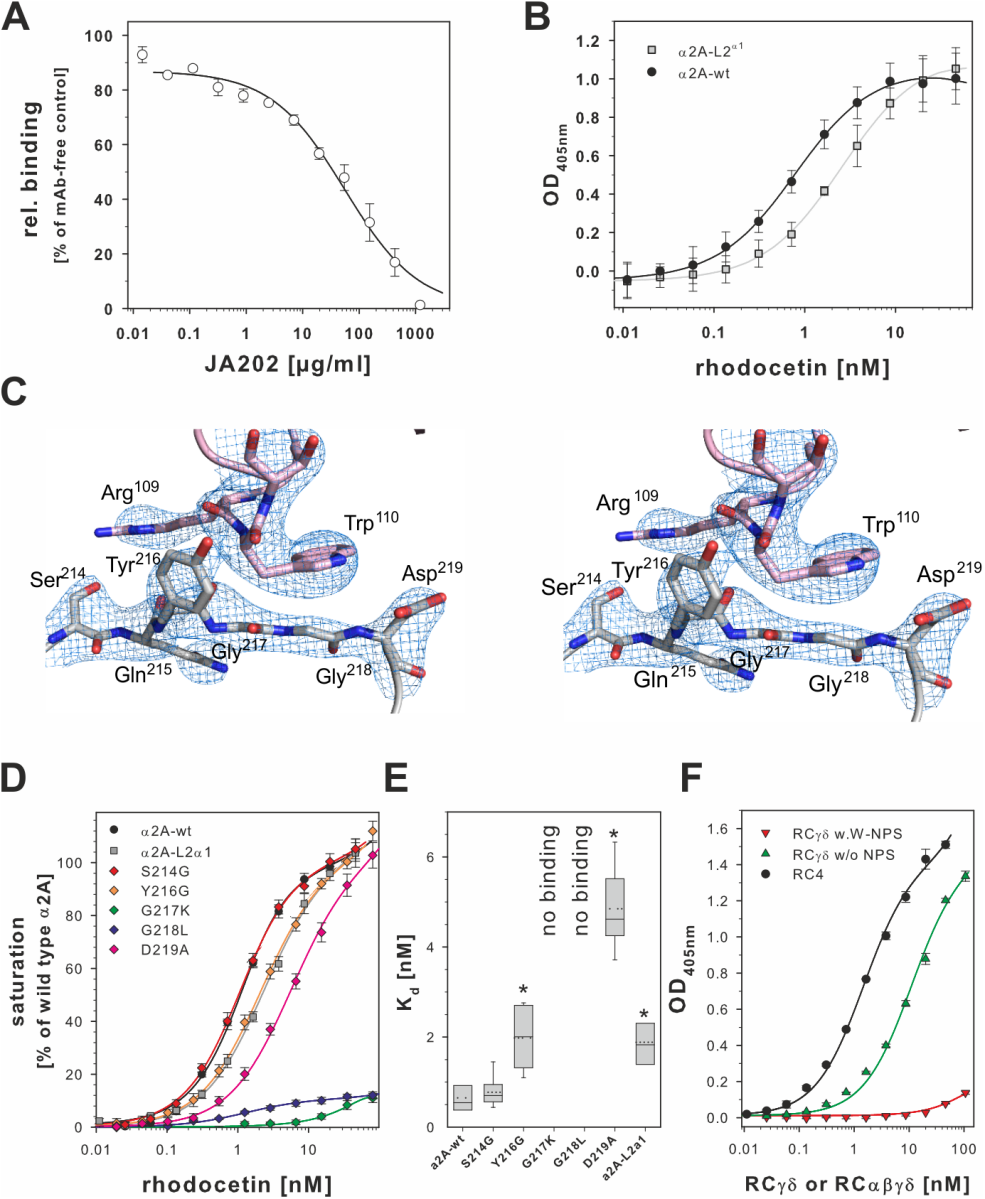
Loop 2 of the α2A domain is the interaction site for the RCγ subunit. (**A**) Loop 2 of α2A is an additional binding site for rhodocetin (RC). It contains the epitope for the monoclonal antibody (mAb) JA202, which inhibits binding of RC to immobilized α2A. Bound RC was quantified by ELISA, and values were normalized to noninhibited controls. One set of inhibition curves out of 3 independent experiments with each measurement made in triplicate and the means ± SD for each data point are shown. (**B**) The α2A loop 2 sequence was replaced with the homologous sequence VGRGGRQ of integrin α1 (α2A L2^α1^ mutant). The binding-irrelevant antibody JA218 was immobilized to capture wild-type (wt) α2A and α2A L2^α1^. They were titrated with RC, and bound RC was quantified as in (**A**). One set of titration curves out of 4 independent experiments, each done in triplicates, is shown with the means ± SD indicated. The α2A L2^α1^ mutant (light gray ■) significantly reduced affinity for RC compared to the wt (●) (*p* = 0.0013, two-tailed *t* test) (**C**) Stereo view of the α2A loop 2 sequence in contact with the RCγ contact site. The Sigma-A weighted 2Fo-Fc map is shown at 1.5σ contour level. The 2 glycine residues, G217 and G218, form the bottom of a shallow dimple, which is flanked on either side by the side chains of Y216 and D219, in addition to residue N154 of loop 1 (not shown). The indole side chain of W110γ stacks directly above this dimple and interacts with the main chain of the 2 glycine residues. (**D**) Point mutation analysis of the α2A loop 2 sequence S^214^QYGGD^219^. The binding activity of these mutants for RC was tested as in (**B**). Binding signals taken from at least 7 independent titration curves for each mutant were normalized to the saturation signal of wild type α2A. Means ± SEM are shown for the mutants (◆ of different colors) in comparison to wt (●) and the α2A L2^α1^ mutant (light gray ■). This analysis showed that the 2 glycines at position 217 and 218 were key to the RCγδ-α2A interaction, as only mutations abrogated α2A binding. (**E**) The K_d_ values of the loop 2 point mutations for binding to RC as derived from (**D**). At least 7 titration curves were evaluated for each mutant. The K_d_ values were pairwise compared to the K_d_ value of the wild type α2A domain in a two-tailed Student *t* test. Significant difference (*p* < 0.02) is asterisked (*). (**F**) Modification of tryptophan residues of RCγδ with 2-nitrophenyl sulfenylchloride (NPS-Cl) showed that W110γ is required for α2A domain binding. The wells of a microtiter plate were coated with 10 μg/ml α2A domain and titrated with RCαβγδ (●), with nonmodified RCγδ (green ▲) and with RCγδ with chemically modified W110γ (W-NPS, red ▼) One representative out 3 independent titration experiments done in duplicate is shown with the means ± SD indicated. The data of plots (**A**), (**B**), (**D**), (**E**), and (**F**) are summarized in S1 Data.

Unlike helix C, the docking site S^214^QYGGD^219^ did not change its conformation between the “open” and “closed” conformation of the α2A domain. To analyze its role, we challenged RC binding to α2A with the monoclonal antibody JA202. Its epitope had previously been mapped to the sequence QTS^214^QY [42] and thus overlapped with the RCγ subunit docking site. Among different antibodies against distinct epitopes within α2A, JA202 was the only monoclonal antibody which sterically inhibited RC binding to the α2A domain in a dose-dependent manner (Fig 7A).

A comparison of integrin α2 chains from different species showed a high interspecies homology of the loop 2 sequence, S^214^QYGGD^219^LT^221^ (S4 Fig). In contrast, this sequence was absent in A-domains of other integrin α subunits, suggesting that it served as a selective docking site for RC on α2β1 integrin (S5 Fig). Therefore, we replaced the α2A sequence S^214^QYGGD^219^L with the corresponding sequence VGRGGRQ of the α1A-domain and tested binding of RC to this α2A-L2^α1^ mutant. Although this α2A mutant was still able to bind RC, the binding affinity was reduced, as indicated by an increase of the K_d_-value from 0.76 ± 0.12 nM to 2.70 ± 0.39 nM (Fig 7B). In parallel to the α2A-L2^α1^ mutant, we exchanged residues in the loop 2 that interacted with RC (Fig 7C), specifically S214, Y216, and D219, as well as the G217 and G218 that are conserved in both integrin α1 and α2 loop 2 sequences, to see which residues were functionally important for the RCγδ-α2A binding. The S214G and D219A mutants, which are located at the outer edges of loop 2, gave K_d_ values of 0.77 ± 0.32 nM and 5.2 ± 1.36 nM, respectively, while the Y216G mutant in the center of the loop gave a K_d_ value of 1.98 ± 0.64 nM (Fig 7D and 7E). In contrast, mutating either of the conserved glycine residues of loop 2 by generating G217K and G218L resulted in a complete loss of RC binding (Fig 7D). This result is in agreement with our structure findings (Fig 7C), which showed that anything larger than a glycine at either position 217 or 218 would sterically clash with the indole side chain of W110γ. In addition, we chemically modified the solvent-exposed W110γ of RC with 2-nitrophenyl sulfenylchloride (NPS-Cl), which introduced a bulky 2-nitro-phenylsulfenyl (NPS) group onto the indole side chain. The modified W110γ is no longer able to stack above the 2 glycines G217 and G218, causing a loss of RC binding to the α2A domain (Fig 7F). Taken together, these results demonstrated that the interaction of W110 of RCγ and the loop 2 of α2A is highly specific and essential for the formation of the high-affinity and inhibitory RCγδ-α2A complex.

## Discussion

Our study reveals not only the interaction sites within RC and its molecular target, the integrin α2A domain, but also the conformational changes that take place within the RCγδ subunit upon α2A binding and the relevance of the 2 contact sites within α2A for RCγδ binding. Moreover, these data suggest a molecular mechanism for the avid and selective interaction of this CLRP and its target.

CLRP dimers recognize other target molecules, such as factor IX/X, and the A-domain of vWF by forming a bay region with their joint index finger loop swap domain and 2 flanking core domains. This concave face shapes the binding sites for clotting factors IX and X [43,44] and the vWF-factor A-domain [45]. Due to their importance in hemostasis, clotting factors and vWF are valid targets for CLRPs from snake venoms. Bitiscetin and botrocetin interact with the vWF–A1 domain without or together with the glycoprotein Ib (GPIb) receptor [27,45,46]. These studies showed that these snake venom toxins can approach the A-domain from different orientations [35,45,46]. In yet another orientation, EMS16 approached the α2A domain of α2β1 integrin, which is homologous to the vWF–A1 domain, along its top face directly above the metal binding site and collagen binding crevice, thus preventing collagen from binding [27]. EMS16 and RC are the 2 α2β1 integrin-binding CLRPs whose crystal structures in both the unliganded and the CLRP in complex with the A-domains have been resolved so far [26,47]. Although RC approached the α2A domain in a similar orientation to EMS16, our data revealed that RC, in contrast to any known CLRP structure [27,45,46], undergoes a dramatic conformational change to form a concave binding surface. In contrast, the heterodimeric EMS16 did not alter its molecular structure upon α2A binding [27,47], as the concave binding surface required for α2A binding was already preformed. This difference in mode of α2A binding between EMS16 and RC is determined by the distinct quaternary structures of the dimeric EMS16 versus the tetrameric RC and/or by the different purification protocols. When we employed the same purification procedure for RC as for EMS16 and other CLRPs [28–30,48] using reversed phase chromatography performed in 0.1% trifluoroacetic acid (TFA) solution, the RC tetramer dissociated into its subunits α, β, and γδ [49]. The RCγδ subunit alone was still able to bind α2A and to block α2β1 integrin-mediated platelet aggregation specifically [50], albeit with a different kinetics [40]. Only when applying a milder purification protocol could we obtain a stable RC tetramer and the RCγδ-α2A complex, whose different conformational structures are presented here.

**Fig 8:**
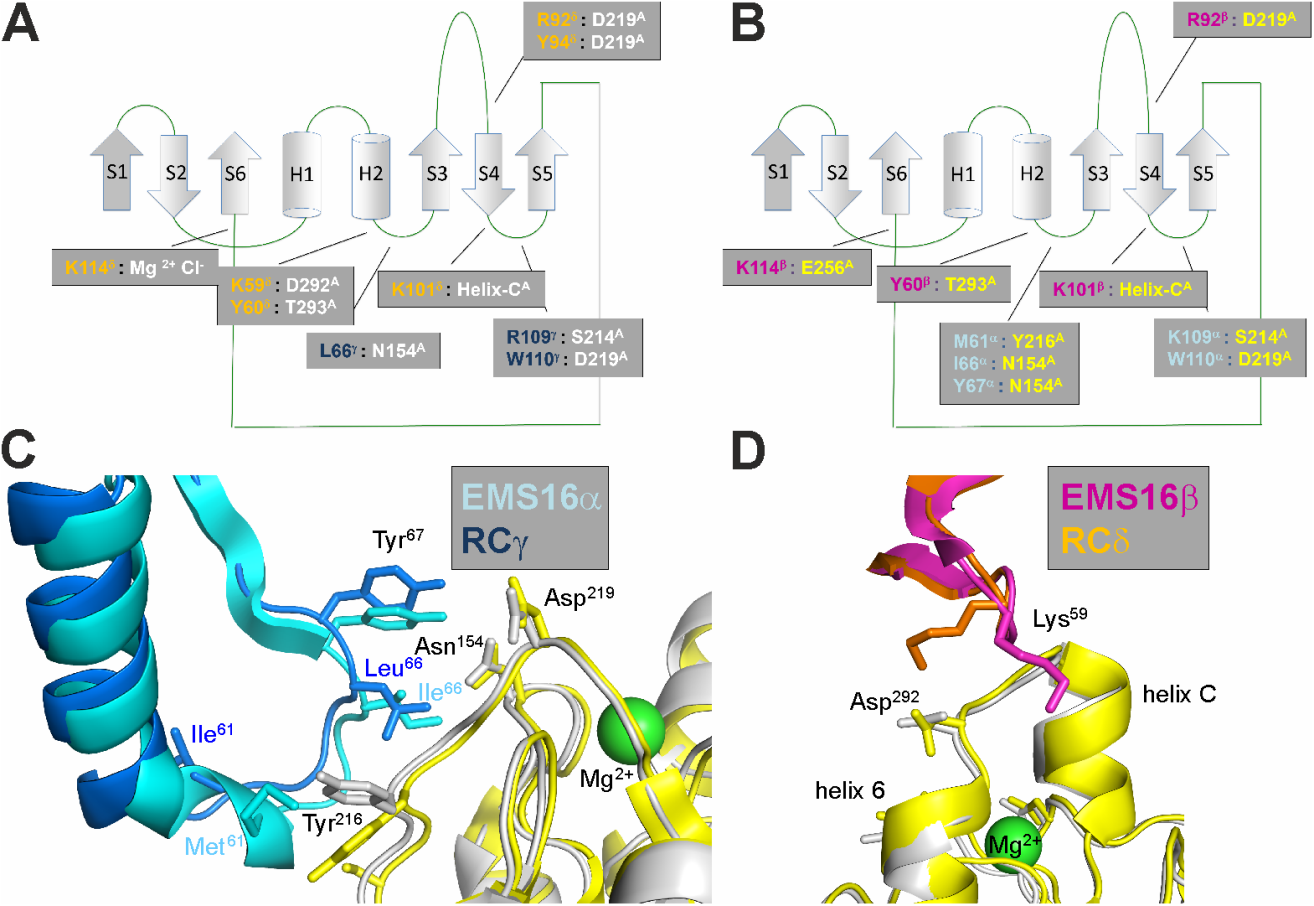
A comparison of the RCγδ-α2A and EMS16αβ-α2A binding interfaces. (**A, B**) The C-type lectin-related protein (CLRP) folds of both homologous subunits of RCγδ (**A**) and EMS16αβ (**B**) are highly homologous with many of the residues involved in the α2A binding conserved between the 2 proteins. These residues have been mapped onto the CLRP fold and colorcoded for rhodocetin (RC) (blue and orange for the γ and δ subunits, respectively, in [**A**]) and for EMS16 (light blue and magenta for the α and β subunits, respectively, in [**B**]). The partnering residues of the α2A domain contacted by RC and EMS16 are color coded in white and yellow, respectively. The same colorcoding scheme is used throughout the figure. (**C, D**) A superposition of the key residues from RCγ/EMS16α at the loop 2 binding site (**C**) and of RCδ/EMS16β at the helix C binding site (**D**), respectively, on α2A. The contact sites are largely conserved between RCγδ/EMS16αβ and α2A, although there are a couple of notable differences. For example, L66 of RCγ contacts Y216 of α2A in addition to the N154 of loop 1 observed for the corresponding I66 of EMS16α. In addition, K59 of RCδ forms a salt bridge to D292 of α2A, whereas, in EMSβ, the corresponding K59 points towards helix C.

Our crystal structure of the RCγδ-α2A complex reveals a geometry of interaction similar to the α2A-bound EMS16, suggesting that the α2β1 integrin-blocking CLRPs may have a more uniform binding mechanism than the vWF binding CLRPs (Fig 8). Both CLRPs share the same 2 contact sites within the α2A domain: the conformationally stable loop 2 sequence (Fig 8C) and the helix C of the “closed” conformation (Fig 8D). Helix C is recognized by the structurally robust contact area of the RCδ subunit or the homologous EMS16 subunit β (or B). Apart from slight variations of the K59δ side chain and the loop 2 Y216 side chain (Fig 8D) adopting an alternate conformation to form a hydrophobic interaction with L66γ, the structures of both complexes are almost identical in this region. In our studies, the role of the loop 2 sequence S^214^QYGGD^219^ was reinforced by the JA202 antibody, whose epitope overlaps with this loop 2 sequence and inhibits RC binding completely, presumably due to steric hindrance by the bulky antibody. More subtly, recombinant exchange of the respective loop 2 sequence with the homologous sequence of integrin α1 showed that the loop 2 sequence changes the affinity of the venom component towards the integrin α2 subunit. Similar reductions in the affinity of RC for α2A were also observed with the loop 2 mutants Y216G and D219A. However, a loss of binding was obtained with the G217K and G218L mutants. These 2 glycine residues form part of a shallow dimple on the α2A surface that is covered by W110 of the RCγ subunit. In the molecular structure of the RCγδ-α2A complex, there is not any space to accommodate anything larger than a glycine at either of these 2 positions, which explains the loss of function of these 2 mutants. The loop 2 sequence of the integrin α2A domain is evolutionary conserved between different animal species, especially the GG motif at positions 217 and 218, but varies remarkably between other integrin α subunits. This suggests that RC’s specificity is mediated by the integrin α2-specific loop 2 sequence, as RC affects α2β1 integrin-mediated platelet blockage in various potential preys but does not affect biological functions mediated by other integrins. Our conclusion—that this cluster of RCγ W110 and G217/G218 of the α2A loop 2 sequence is a key to the RCγδ-α2A interaction—is further supported by the fact that the RC binding is completely lost if the bulky chemical adduct of 2-nitrophenylsulfenyl is introduced to the indole side chain. It is noteworthy that the loop 2 sequence is also relevant for collagen binding, as it forms a hydrophobic contact for the phenylalanine side chain of the middle strand of the trimeric integrin recognition motif of collagen [15], albeit not as close a contact as with the RCγ W110 side chain.

**Fig 9:**
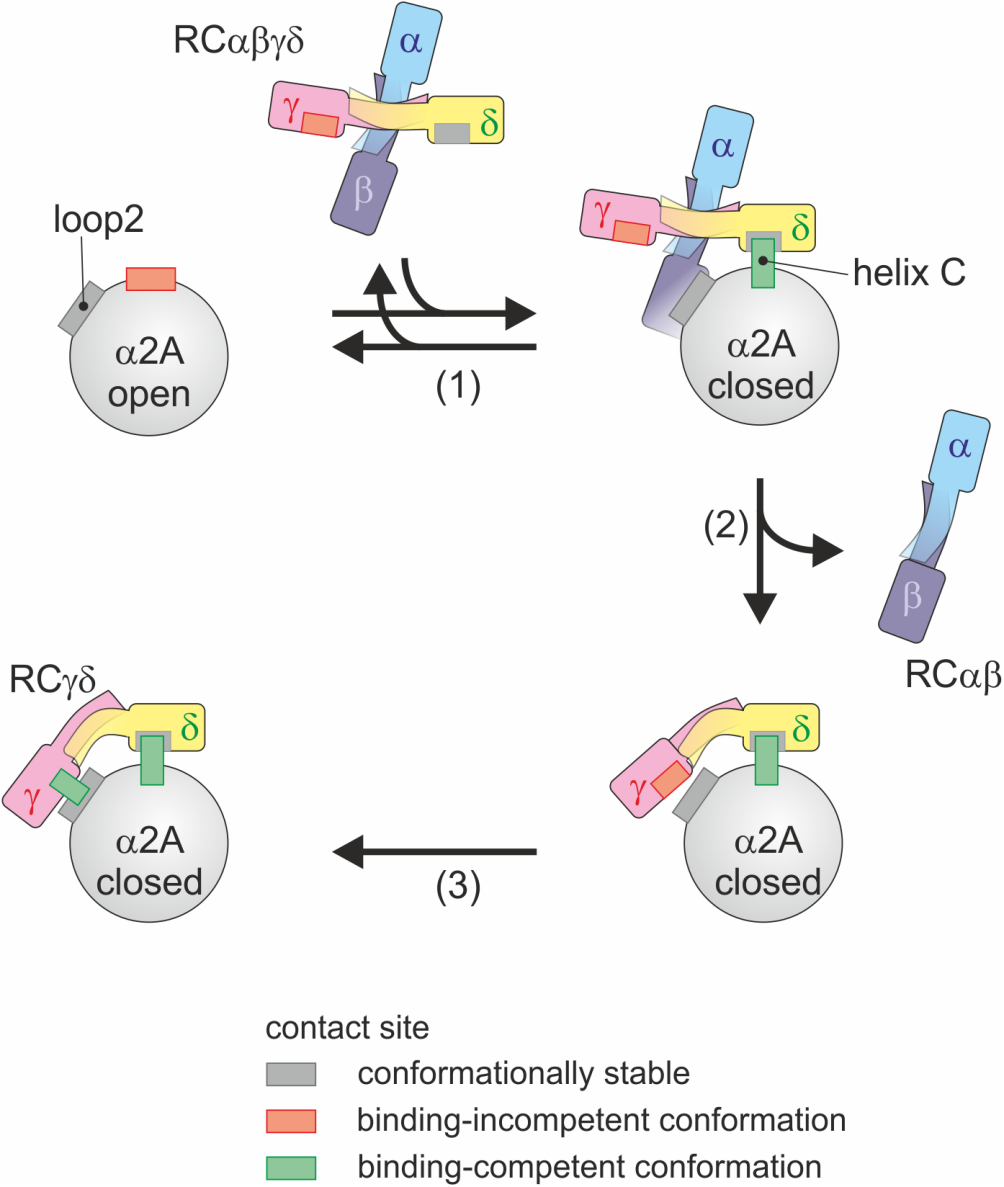
Molecular mechanism of the RCγδ-α2A interaction. As RCαβγδ binds to α2A in its “closed” conformation, it induces the conformational change of α2A from its “open” to “closed” conformation and thus shifts the conformational equilibrium (1). This interaction is mediated via the conformationally robust RCδ interaction site within helix C, which is only present in the “closed” conformation of α2A. Subsequently, the index finger loop of RCγ changes its conformation, which is accompanied by a global movement of both rhodocetin (RC) core domains towards each other and by a release of the RCαβ subunit (2). As the RCαβ subunit diffuses away, this step is likely irreversible in nature. The global shape change of RCγδ forms a new bay region that embraces α2A and locally leads to the repositioning of RCγ key residues, which forms another binding-competent interacting site in RCγ for the α2A loop 2 (3).

Based on our findings, we suggest the following mode of action (Fig 9). RCαβγδ interacts with helix C of the α2A domain through the RCδ subunit, where the interacting residues are already in binding-competent orientation. This stabilizes the “closed” conformation of α2A. As a consequence of the movement of RCγ, the RCαβγδ tetramer changes conformation such that RCαβ dissociates from the heterotetrameric assembly. Coupled to this dissociation is the reorganization of L66, R109, and W110 of RCγ to interact with loop 2 sequence S^214^QYGGD^219^. Having established both interaction sites, RCγδ firmly binds to α2A and holds it in the “closed” conformation, thereby blocking collagen binding and antagonistically turning off α2β1 integrin signaling. After its release upon formation of the high-affinity RCγδ-α2β1 complex, the RCαβ subunit plays another important role in blocking GPIb and, consequently, vWF-induced platelet aggregation [49]. Moreover, our biochemical data showed that the RCαβ subunit is significantly more soluble than the RCγδ subunit [40]. Therefore, it likely acts as a solubility enhancer to ensure that the RCγδ subunit is delivered to α2β1 integrin. Once RCγδ has bound to its target and the RCαβ subunit has been released, RC effectively shuts down the 2 platelet receptors, α2β1 integrin and GPIb, thereby effectively blocking both collagen-induced and vWF-induced platelet activation and aggregation.

In summary, a comparison of the RCγδ-α2A structure with the EMS16-α2A integrin complex [27] shows that the residues involved in the binding of RC and EMS16 to α2β1 integrin are highly conserved. The formation of the inhibitory RC-α2A complex requires both the interaction of RCδ with the helix C of α2A and RCγ with the α2A loop 2 sequence. Furthermore, the presence of helix C in our structure confirms that we have trapped α2A in the “closed” conformation, which is not able to bind collagen and explains why RC is able to block collagen-mediated platelet aggregation. Finally, the requirement of 2 separate sites within the α2A domain for both function and specificity may be instrumental for the design of novel α2β1 integrin inhibitors.

## Materials and methods

### Materials

RC and its γδ subunit were isolated as previously described [40,51]. The monoclonal antibodies (mAbs) against RC, among them IIIG5 from mice and IC3 from rats, were generated and isolated as previously described [40]. The murine mAbs against the human α2A domain, JA202 and JA218, were a generous gift from D. Tuckwell (formerly of the University of Manchester, United Kingdom) [40,42]. PCR primers were obtained from Eurofins (Eurofins Genomics, Germany) and are written in 5′-3′ direction. Restriction enzymes and molecular biology reagents were from Thermo Fisher Scientific (Germany) unless otherwise stated. Cloning products and expression vectors were validated by DNA sequencing (Eurofins Genomics).

### Tryptophan-specific chemical modification of RC

RC, dissolved at 110 μM in 30% acetic acid solution, was treated with 9.2 mM 2-nitrophenyl sulfenylchloride (NPS-Cl, TCI Chemicals, Germany) or left untreated for 1 h at 20 °C in the dark according to [52], subsequently dialyzed against 0.1% TFA (RP-solution) and separated on a Supercosil C18 column (Supelco, Germany) by reversed-phase chromatography as described [26]. The RCγδ-containing fractions were pooled, lyophilized, and dissolved in RP-solution containing 30% acetonitrile. Purity was assessed by SDS-PAGE. Spectroscopic evaluation at 365 nm according to [52] confirmed the covalent modification of RC tryptophan residues with 2-nitro-phenylsulfenyl (NPS)-groups.

### Isolation of RCγδ-α2A complex

The His_6_-tagged α2A domain was generated as previously described [26,53]. It was loaded onto a HiTrap Ni Sepharose column (GE Healthcare; 5 ml) previously equilibrated with PBS/MgCl_2_-buffer, pH 7.4 (20 mM sodium phosphate, pH 7.4, 150 mM NaCl, 1 mM MgCl_2_). After washing with the same buffer, the RCαβγδ-containing fractions from the RC isolation with MonoS column [51] were applied to the α2A domain loaded Ni Sepharose column after having been treated with 0.5 μM phenylmethylsulfonyl fluoride (PMSF) and 1 μg/ml aprotinin to prevent proteolytic digestion by potentially contaminating snake proteases. After RCαβγδ had bound to the Ni Sepharose-immobilized α2A domain, the HiTrap Ni Sepharose column was washed with PBS/MgCl_2_-buffer, pH 7.4. Then, the column was washed with PBS/EGTA-buffer, pH 7.4 (5 mM EGTA in 20 mM sodium phosphate, pH 7.4, 150 mM NaCl) and the RCαβ subunit eluted. After another washing step with PBS/MgCl_2_-buffer, pH 7.4, the RCγδ-α2A complex was eluted with a linear gradient of 0–200 mM imidazole in PBS/MgCl_2_-buffer, pH 7.4 from the HiTrap Ni Sepharose column. Protein concentration in the imidazole eluate was determined using the Bradford reagent (BioRad). For crystallization, the complex-containing fractions were pooled and digested with TPCK-treated trypsin (Sigma-Aldrich) at an enzyme:substrate ratio of 1:100 at 37 °C for 1 h. The digest was stopped with 1 mM PMSF, concentrated and separated by gel filtration to remove excess α2A domain, trypsin and contaminating peptides from the RCγδ-α2A complex. The TSK G2000SWXL chromatography was performed in 10 mM HEPES, pH 7.4, 100 mM NaCl buffer. The RCγδ-α2A complex was concentrated by ultrafiltration and its protein concentration determined with the Bicinchoninic Acid Protein Assay (BCA, Thermo Fisher Scientific). To analytically prove the physical contact of both partners, the complex was cross-linked with 0.5 mM bi-sulfosuccinimidyl-suberate (BS^3^, Thermo Fisher Scientific). Its IEP was determined to be pH 6.5–6.8 and pH 6.7 by isoelectric focusing in precast ZOOM pH 3–10 gels (Thermo Fisher Scientific) and by analytical chromatofocusing on a MonoP column (GE HealthCare) with a pH gradient of 7.4 to 4.0, respectively.

### Crystallization, data processing, and structure refinement

Crystals of 10 mg of RCγδ-α2A were grown by hanging-drop vapor diffusion at 293 K by mixing 2 μL of protein solution with 2 μL reservoir solution containing 2.65 M ammonium sulfate and 100 mM Tris pH 8.0. Crystals appeared after 6 weeks and were soaked in mother liquor containing 20% glycerol for 5–10 min before being flash frozen in liquid nitrogen. Diffraction data was collected at the Canadian Light Source CMCF-08ID-1 beamline (λ = 0.97949Å) at 100 K using a Rayonix MX225 CCD detector. The dataset was indexed, integrated, and scaled with MOSFLM [54] and the CCP4-package [55]. The spacegroup is P4_1_ with 6 molecules in the asymmetric unit (see also Table 1). The phases were determined by rigid body refinement using the previously solved RC structure (PDB code 3GPR) in Refmac [56,57]. The model was built and refined without NCS restraints using Coot [58] and refined with the Phenix software package [59]. The crystallographic data and refinement statistics are summarized in Table 1. The final coordinates and structure factor amplitudes were deposited in the PDB (RCSB-code: 5THP).

**Table 1:** Data and refinement statistics of the RCγδ-α2A crystal structure.

Data collection	RCγδ-α2A complex
λ (Å)	0.97949
Space Group	P4_1_
Cell dimensions	
*a*, *b*, *c* (Å)	130.763, 130.763, 251.351
*α*, *β*, *γ* (°)	90.00, 90.00, 90.00
No. reflections^a^	438487 (22219)
Resolution (Å)	19.87–3.01 (3.06–3.01)
*R*_*merge*_	0.096 (0.607)
*I/*σI	11.9 (2.3)
Completeness (%)	99.3 (93.2)
Multiplicity	5.3 (5.2)
**Refinement**	
*R*_*work*_*/R*_*free*_	0.2182/0.2715
No. atoms	
Protein	20614
Ligand/Ion	83
Water	254
*B-*factor (Å^2^) Protein/Water	79.17/63.74
R.m.s. deviations	
Bond lengths (Å)	0.007
Bond angles (°)	0.669

^a^ Statistics of the highest resolution shell are shown in parentheses

### Generation of integrin α2A domain mutants

The human α2A domain and its mutants were produced in a bacterial expression system. The expression vectors encoding the disulfide-locked conformation mutants of α2A were generated using a previously described pET15b-His_6_-α2A construct (residues 142 through 337 of human integrin α2). To replace the endogenous cysteine residues at 150 and 270, this plasmid was used as template for a 2-step PCR with the 3 primer pair sets (i) HT*fwd*(CTCTCCATGGGCTCTTCTCATCATCATCATCATCATTC) and R1(C11A) (CATC**AGC**CACAACCACAAC), (ii) F2(C11A) (TTGTG**GCT**GATGAATCAAATAG) and R2(C131A) (TTG**GCT**TGATCAATCACAGC), and (iii) F3(C131A) (ATTGATCAA**GCC**AACCATGAC) and α2A*rev* (CGGACATATGCTAACCTTCAATGCTGAAAAATTTG) in the first set of reactions. The 3 amplicons were purified and again PCR-amplified with the outer primer pair HT*fwd* and α2A*rev* to a 670 bp amplicon, which, after A-tailing with Taq DNA polymerase, was intermediately ligated into pCR2.1 TOPO, excised with *Nde*I and *Nco*I, and the restriction fragment was subcloned into the linearized, *Nde*I, *Nco*I-cleaved pET-15b expression vector. The final expression plasmid pET-15b-His_6_-α2A(C150,270A) was transformed into *Escherichia coli* BL21 (DE3).

To generate the disulfide-locked conformation mutants of α2A, which share the same K168C mutation but differ in E318C (“open” conformation: K168C, E318C) or A325C (“closed” conformation: K168C, A325C), 3 rounds of PCR amplification were performed. In the first, site-directed mutagenesis K168C was introduced by amplifying the entire plasmid with the back-to-back primer pair K168C fw (AAGGCCTGGATATAGGCCCC) and K168C rev (GTACAAAGCATTCCAAAAAATTCTTTACTGC). Based on this mutation, the final 2 mutants (K168C, E318C; K168C, A325C) were similarly generated using the primer pairs E318C fw (GTCTGATTGCGCAGCTCTACTAGAAAAG)/E318C rev (ACATTGAAAAAGTATCTTTCTGTTGGAATAC) and A325C fw (ATTAGGAGAACAAATTTTCAGCATTGAAG)/A325C rev (GTCCCGCACTTTTCTAGTAGAGCTG). For each site-directed mutagenesis, only 1 primer contained the specific mutation. The PCR products were amplified by the Phusion Hot Start II polymerase and covered the whole template vector (6307 bp) with the mutation. After the original, methylated vector had been digested with *DpnI*, the amplicons were purified using the DNA Clean & Concentrator Kit (Zymo Research), followed by 5′-phosphorylation with T4 polynucleotide kinase and religated using T4 DNA ligase. For protein expression, *E*. *coli* strain BL21 (DE3) were transformed with the validated plasmid constructs encoding the α2A domain in its “open” (pET-15b-His_6_-α2A-C150/270A-K168C/E318C) and “closed” (pET-15b-His_6_-α2A-C150/270A-K168C/A325C) conformations.

The α2A-L2^α1^ mutant, in which the sequence S^214^QYGGDL is replaced by the corresponding loop 2 sequence V^214^QRGGRDQ of the integrin α1 A-domain, was generated by 2-step PCR. The pET15b-construct encoding the His-tagged α2A domain [26] was used as a template. The primer pairs α2A fw (GGATATCTGCAGAATTCGCCCTTC) and R1_a1insert into a2 (CTTTACTAACATCGTTGTAGGGTCTGTCACGTCGCGCCACCAGCGGTC), F1_a1insert into a2 (GTGCAGCGCGGTGGTCGCCAGACAAACACATTCGGAGCAATTC), and α2A rev (AGGCCATATGCTAACCTTCAATGCTGAAAATTTG) amplified the N- and C-terminal halves of the cDNA. The 2 amplicons were mixed and amplified with the outer primer pair. The resulting 680 bp amplicon was trimmed with *Nco*I and *Nde*I, ligated into a correspondingly cut pET-15b vector, verified by sequencing, and transformed into *E*. *coli* BL21(DE3).

Point mutations within the loop 2 sequence were also generated by a 2-step PCR using the wild-type α2A-encoding cDNA as template. First, cDNA fragments encoding the N- and C-terminal halves of α2A were amplified by using the 2 pairs of forward outer and reverse inner primers and of forward inner and reverse outer primers, respectively, as summarized in Table 2.

**Table 2:** PCR primers for cloning the α2A loop2 mutants.

**Outer primers:**	
Forward outer primer: (NdeI site underlined)	5′-GCAGCCATATGGGAGGTTCTCCTTCCCTCATAGATGTTGTGGTTGTG-3′
Reverse outer primer: (BamHI site underlined)	5′-AGCCGGATCCTCGAGCTACTAACCTTCAATGCTGAAAATT TGTTC-3′
**Inner primers:** (mutation sites are underlined)	
S214A-forward:	5′-GCAACATCCCAGACAGGTCAATATGGTGGGG-3′
S214A-reverse:	5′-CCCCACCATATTGACCTGTCTGGGATGTTGC-3′
Y216G-forward:	5′-CCCAGACATCCCAAGGTGGTGGGGACCTCAC-3′
Y216G-reverse:	5′-GTGAGGTCCCCACCACCTTGGGATGTCTGGG-′
G217K-forward:	5′-CAGACATCCCAATATAAAGGGGACCTCACAAAC-3′
G217K-reverse:	5′-GTTTGTGAGGTCCCCTTTATATTGGGATGTCTG-3′
G218L-forward:	5′-GACATCCCAATATGGTCTGGACCTCACAAACAC-′
G218L-reverse	5′-GTGTTTGTGAGGTCCAGACCATATTGGGATGTC-3′
D219A-forward:	5′-CAATATGGTGGGGCACTCACAAACACATTCGGAGC-3′
D219A-reverse:	5′-GCTCCGAATGTGTTTGTGAGTGCCCCACCATATTG-3′

The amplicons were purified and taken as template for a second PCR with the outer primer pair to obtain the wild-type and mutant α2A domains encoding cDNAs, which were digested with *NdeI* and *BamHI* and ligated into the likewise-cut pET-15b vector. After verification by sequencing, the expression vectors were transformed into *E*. *coli* BL21(DE3). All α2A domain mutants were purified using HiTrap Ni Sepharose column (GE HealthCare) as per the wild type.

### Binding and inhibition assays of α2A domain with RC

The wells of a half-area microtiter plate (Costar) were coated with 10 μg/ml His-tagged α2A domain in TBS/Mg buffer (50 mM Tris/HCl, pH 7.4, 150 mM NaCl, 3 mM MgCl_2_) at 4 °C overnight. After washing twice with TBS/Mg buffer, the wells were blocked with 1% BSA in TBS, pH 7.4, 2 mM MgCl_2_ for 1 h at room temperature. The immobilized α2A domain was titrated with a serial dilution of RCαβγδ or RCγδ without and with NPS-modified tryptophans in the blocking buffer for 1.5 h. For the mAb inhibition experiment, RC at a constant concentration of 2 nM was added to the wells in either the absence or presence of mAb JA202 against RC. After washing twice with HEPES-buffered saline (HBS) (50 mM HEPES/NaOH, pH7.4, 150 mM NaCl, 2 mM MgCl_2_), bound RC was fixed with 2.5% glutaraldehyde in the same solution for 10 min at room temperature. After 3 additional washes with TBS/Mg buffer, bound RC was quantified by ELISA using a primary rabbit antiserum against RC and a secondary alkaline phosphatase conjugated anti-rabbit–IgG antibody, each diluted 1:2,000 in 1% BSA/TBS/Mg. Conversion of para-nitrophenyl phosphate (pNpp) to para-nitrophenolate was stopped with 1.5 M NaOH and measured at 405 nm. The titration curves were evaluated as described below. The inhibition curves were approximated by GraphPad Prism software using the inhibition vs. log [inhibitor]-approximation. To compare independent inhibition and binding experiments, the dynamic ranges were normalized to the mAb-free control and to the saturation value of the wild-type α2A domain, respectively.

Alternatively, the α2A domains, either wild-type or mutants, were captured using the mAb JA218 at a ligand-binding–irrelevant epitope, thereby avoiding any conformational changes due to adsorption to the plastic. To this end, 2.5 μg/ml JA218 was immobilized to a microtiter well at 4 °C overnight. After the wells were washed twice with TBS/Mg buffer, wells were blocked with 1% BSA in the same buffer for 1 h, and then, the α2A domain was added at 10 μg/ml for 1 h. After washing the wells, RC was titrated and detected as described above.

### Capturing ELISA with IIIG5

The mAb IIIG5 was coated to the wells of a microtiter plate at 3 μg/ml in TBS/Mg buffer overnight. After 2 washing steps, wells were blocked with 1% BSA in TBS/Mg buffer for 1 h and then titrated with either RCαβγδ, RCγδ, or RCγδ-α2A complex for 1.5 h at room temperature. Bound RC was fixed and quantified as described above. A mathematical approximation of the titration curve, including determination of K_d_-values, is described below.

### Isolation of IIIG5 epitope and mass spectrometry

IIIG5 was immobilized to cyanogen bromide-activated sepharose according to the manufacturer’s instruction (GE Healthcare). RCαβγδ-containing fractions from the Mono S purification of *C*. *rhodostoma* venom [26] were reduced with 4 mM tris(hydroxymethyl)phosphine (THP, Calbiochem) for 20 min at 60 °C, and free thiol groups were alkylated with 16 mM iodoacetic acid. The protein was precipitated with trichloroacetic acid, washed with acetone twice, resuspended in 87.5 mM sodium bicarbonate/0.5 M urea and digested with TPCK-trypsin for 23 h at 37 °C. After addition of 1 mM PMSF, the digest was diluted with TBS/HCl buffer, pH 7.4 and loaded onto the IIIG5 column. The RC peptide harboring the IIIG5 epitope was eluted in a pH gradient from pH 7.5 to 3.0 in 20 mM citrate buffer and further purified by reversed phase on a Supercosil C18 column in a 0%–28% acetonitrile gradient in 0.1% TFA/water. Lyophilized HPLC fractions were dissolved in 40% methanol containing 0.5% formic acid and analyzed by nano-electrospray ionization (nanoESI) MS and MS/MS. Peptide structures were deduced from the corresponding fragment ion spectra. NanoESI MS experiments were carried out by using a SYNAPT G2-S mass spectrometer (Waters, Manchester, UK) equipped with a Z-spray source in the positive ion sensitivity mode. Typical source parameters were as follows: source temperature, 80 °C; capillary voltage, 0.8 kV; sampling cone voltage, 20 V; and source offset voltage, 50 V. For low-energy collision-induced dissociation (CID) experiments, the peptide precursor ions were selected in the quadrupole analyzer, subjected to ion mobility separation (IMS; wave velocity 850 m/s, wave height 40 V, nitrogen gas flow rate 90 ml/min, and helium gas flow rate 180 ml/min), and fragmented in the transfer cell using a collision gas (Ar) flow rate of 2.0 ml/min and collision energies up to 100 eV (*E*_lab_).

### Mathematical evaluation of titration curves

In titration curves, a signal S, usually the extinction at 405 nm caused by the alkaline phosphatase-catalyzed conversion of pNpp, is measured in response to the total concentration c_0_ of added titrant. Based on a Michaelis–Menten-like binding mechanism, we deduced the following equation to approximate titration curves, if the signal S and the total concentration c_0_ of added ligand (RC) is known:

$$S(c_{0})=(S_{M}-S_{m})∙\left(\frac{(c_{0}+c_{R}+K)-\sqrt{{\left(c_{0}+c_{R}+K\right)}^{2}-4∙c_{0}∙c_{R}}}{2∙c_{R}}\right)+S_{m}+B∙c_{0}$$

with S_M_ and S_m_, maximum and minimum signals, respectively; c_R_, the concentration of ligand binding site (equals the receptor concentration for monovalent receptors); and K, the dissociations constant K_d_. The term B·c_0_ takes into account a linear change in the signal due to nonspecific binding of the ligand. The 5 parameters S_M_, S_m_, c_R_, K, and B are calculated by nonlinear regression from titration curves.

### Statistical analysis

The data from titration and inhibition curves were statistically evaluated using GraphPad Prism software. Values were usually compared with the values obtained for the wild-type α2A or nonmodified RC with Student *t* test, where the significance level was set at 1% unless otherwise stated.

## Supporting information

S1 Fig Asymmetric unit of the RCγδ-α2A crystal structure. (**A**) Overall view of the asymmetric unit showing six RCγδ-α2A complexes. Individual α2A domains are shown in grey, with the Mn^2+^ as pink spheres. RCγ subunits are shown in red, whereas RCδ subunits are in yellow. (**B**) The different heterotrimeric assemblies can be subcategorized in three different interaction modes. Domain-domain contacts are mediated either via the core segment of the CLRP fold of RCγ (top), the distal end of the α2A domain (middle) or the index finger loop segments (bottom). Remarkably, the overall r.m.s.d. in Cα positions for all individual subdomains is 1.1Å demonstrating that the different RCγδ-α2A complexes are identical.
(TIF)

S2 Fig Molecular model of the disulfide-locked conformation mutants of α2A domain. By introducing disulfide bridges at the respective sites, helices 1 and 7 were fixed towards each other. Using this approach, the α2A domain is stabilized in either the “open” or “closed” conformation. (**A**) Model of K168C-E318C representing the open conformation. (**B**) Model of K168C-A325C showing the conformation. Residues involved in the formation of helix C are in red. To highlight the difference between the two conformations, amino acid residue positions 318 and 325 are colored blue and green, respectively. Structures were modelled with Pymol using the pdb data sets of α2A domain in its “open” (1DZI) and “closed” (1AOX) conformation.
(TIF)

S3 Fig Identification of the IIIG5 epitope within the RCγ chain. **(A**) Fragmentation scheme for the tryptic peptide, of the RCγ subunit containing the IIIG5 epitope. (**B) **NanoESI fragment ion spectrum of the RCγ peptide containing the IIIG5 epitope. It was obtained from a CID experiment on the ion mobility-separated doubly charged peptide precursor ions at m/z 942.40. The labelled peaks correspond to the fragment ions of this epitope peptide, as shown in (**A**).
(TIF)

S4 Fig Alignment of integrin α2A domains from different species. Sequence comparison of the integrin α2 A-domain from different vertebrate species. The loop 2 sequence S^214^QYGGD is highlighted in yellow and shows a high degree of homology between different species. Multiple sequence alignment was carried out with Clustal Omega Software from EMBL-EBI.
(TIF)

S5 Fig Alignment of A-domain of different human integrin α-chains. A comparison of A-domains from different human integrin α subunits. Integrin alpha subunits 1, 2, 10, and 11 belong to the subset of collagen binding integrins. They possess the characteristic helix C (yellow box, labelled α-C), which is absent in the A-domain of the leukocyte β2 integrins with their alpha subunits L, X, M, and D. Helix C of the integrin α2 subunit is the primary binding site for RCγδ and is only present in the “closed” conformation of its A domain. The secondary RC contact site of α2A is located within the loop 2 sequence S^214^QYGGD, (yellow box, labelled loop 2) and is specific to the integrin α2 chain. The secondary structure elements are indicated by the red (α-helices) and the blue (β-strands) boxes, respectively. The residue numbering refers to the integrin α2 sequence. Multiple sequence alignment was carried out with Clustal Omega Software from EMBL-EBI.
(TIF)

S1 Data Summary of data of Figs 3A, 4A, 7A, 7B, 7D, 7E and 7E. (XLSX)

## Abbreviations

BSA: bovine serum albumin
CLRP: C-type lectin-related protein
CRD: carbohydrate recognizing domain
ECM: extracellular matrix
GPIb: glycoprotein Ib
HPLC: high-performance liquid chromatography
MS: mass spectrometry
NPS: 2-nitro-phenylsulfenyl
NPS-Cl: 2-nitrophenyl sulfenyl chloride
RC: rhodocetin
TFA: trifluoroacetic acid
vWF: von Willebrand factor
