# Correction: Dramatic and concerted conformational changes enable rhodocetin to block α2β1 integrin selectively

**DOI:** 10.1371/journal.pbio.1002613

**Published:** 2017-09-06

**Authors:** Johannes A. Eble, Matthew McDougall, George L. Orriss, Stephan Niland, Benjamin Johanningmeier, Gottfried Pohlentz, Markus Meier, Simone Karrasch, Maria Inacia Estevão-Costa, Augusto Martins Lima, Jörg Stetefeld

The supplementary figures were incorrectly allocated and labelled:

- S1 Fig was mistakenly labelled and described as S5 Fig
- S2 Fig was mistakenly labelled and described as S1 Fig
- S3 Fig was mistakenly labelled and described as S2 Fig
- S4 Fig was mistakenly labelled and described as S3 Fig
- S5 Fig was mistakenly labelled and described as S4 Fig

The authors have provided the correctly labelled supplementary figures. The legends remain the same.

## Supporting information

S1 Fig Asymmetric unit of the RCγδ-α2A crystal structure. (A) Overall view of the asymmetric unit showing six RCγδ-α2A complexes. Individual α2A domains are shown in grey, with the Mn^2+^ as pink spheres. RCγ subunits are shown in red, whereas RCδ subunits are in yellow. (B) The different heterotrimeric assemblies can be subcategorized in three different interaction modi. Domain-domain contacts are mediated either via the core segment of the CLRP fold of RCγ (top), the distal end of the α2A domain (middle) or the index finger loop segments (bottom). Remarkably, the overall r.m.s.d. in Cα positions for all individual subdomains is 1.1Å demonstrating that the different RCγδ-α2A complexes are identical.
(JPG)

S2 Fig Molecular model of the disulfide-locked conformation mutants of α2A domain. By introducing disulfide bridges at the respective sites, helices 1 and 7 were fixed towards each other. Using this approach, the α2A domain is stabilized in either the “open” or “closed” conformation. (A) Model of K168C-E318C representing the open conformation. (B) Model of K168C-A325C showing the conformation. Residues involved in the formation of helix C are in red. To highlight the difference between the two conformations, amino acid residue positions 318 and 325 are coloured blue and green, respectively. Structures were modeled with Pymol using the pdb data sets of α2A domain in its “open” (1DZI) and “closed” (1AOX) conformation.
(JPG)

S3 Fig Identification of the IIIG5 epitope within the RCγ chain. (A) Fragmentation scheme for the tryptic fragment of the RCγ subunit containing the IIIG5 epitope. (B) NanoESI fragment ion spectrum of the RCγ peptide containing the IIIG5 epitope. It was obtained from a CID experiment on the ion mobility-separated doubly charged peptide precursor ions at m/z 942.40. The labelled peaks correspond to the fragmentation ions of this epitope peptide, as shown in (A).
(JPG)

S4 Fig Alignment of integrin α2A domains from different species. Sequence comparison of the integrin α2 A-domain from different vertebrate species. The loop 2 sequence S214QYGGD is highlighted in yellow and shows a high degree of homology between different species. Multiple sequence alignment was carried out with Clustal Omega Software from EMBL-EBI.
(JPG)

S5 Fig Alignment of A-domain of different human integrin α-chains. A comparison of A-domains from different human integrin α subunits. Integrin alpha subunits 1, 2, 10, and 11 belong to the subset of collagen binding integrins. They possess the characteristic helix C (yellow box, labelled α-C), which is absent in the A-domain of the leukocyte β2 integrins with their alpha subunits L, X, M, and D. Helix C of the integrin α2 subunit is the primary binding site for RCγδ and is only present in the “closed” conformation of its A domain. The secondary RC contact site of α2A is located within the loop 2 sequence S214QYGGD, (yellow box, labelled loop 2) and is specific to the integrin α2 chain. The secondary structure elements are indicated by the red (α-helices) and the blue (β-strands) boxes, respectively. The residue numbering refers to the integrin α2 sequence. Multiple sequence alignment was carried out with Clustal Omega Software from EMBL-EBI.
(JPG)
