# Supplementary figures and images for: Dramatic and concerted conformational changes enable rhodocetin to block α2β1 integrin selectively
Source: PLoS Biol. 2017 Jul 13;15(7):e2001492. doi: 10.1371/journal.pbio.2001492 (PMC5509089; doi:10.1371/journal.pbio.2001492)

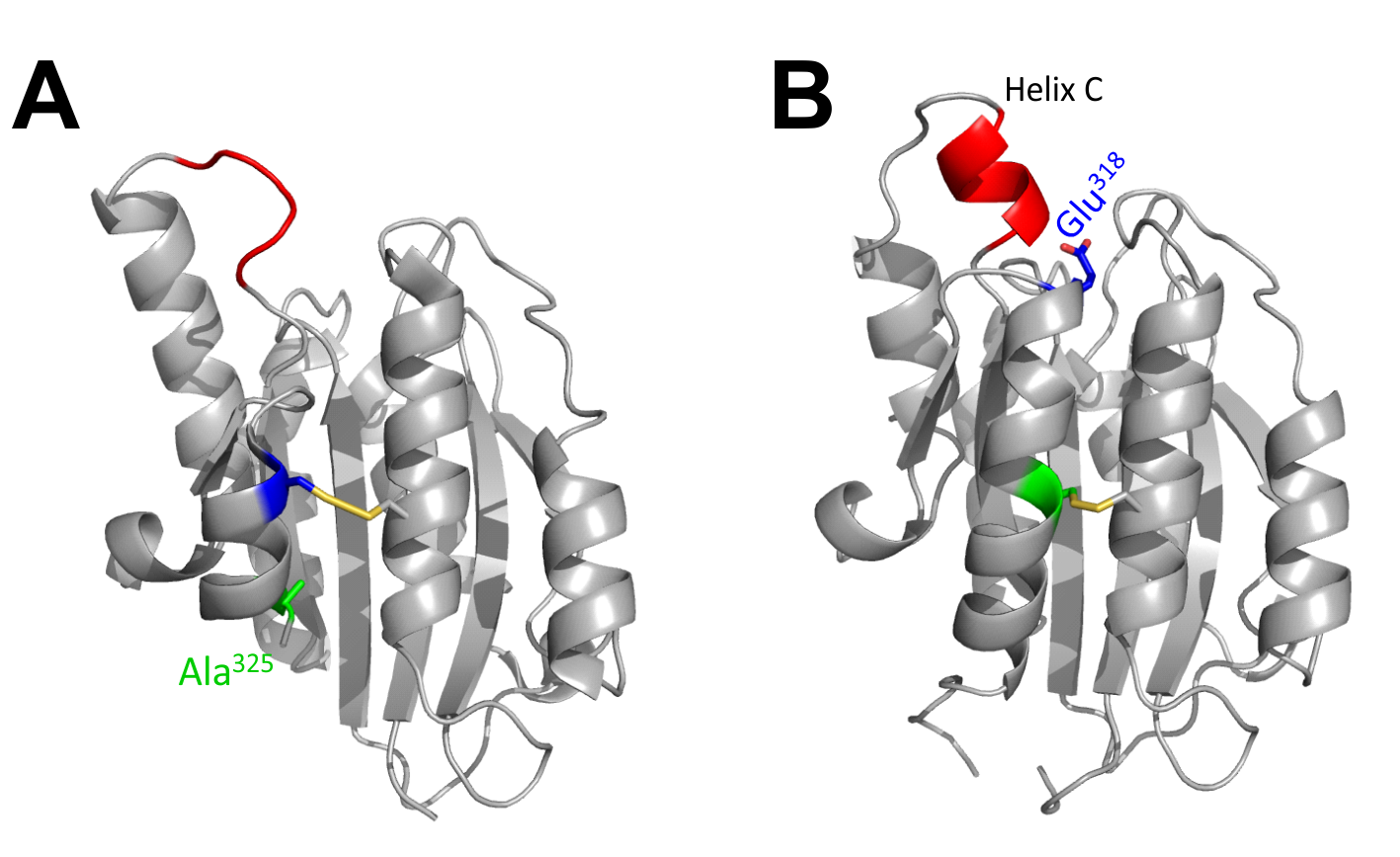

Supplement: S1 Fig — (A) Overall view of the asymmetric unit showing six RCγδ-α2A complexes. Individual α2A domains are shown in grey, with the Mn2+ as pink spheres. RCγ subunits are shown in red, whereas RCδ subunits are in yellow. (B) The different heterotrimeric assemblies can be subcategorized in three different interaction modes. Domain-domain contacts are mediated either via the core segment of the CLRP fold of RCγ (top), the distal end of the α2A domain (middle) or the index finger loop segments (bottom). Remarkably, the overall r.m.s.d. in Cα positions for all individual subdomains is 1.1Å demonstrating that the different RCγδ-α2A complexes are identical. (TIF) [file pbio.2001492.s001.tif]

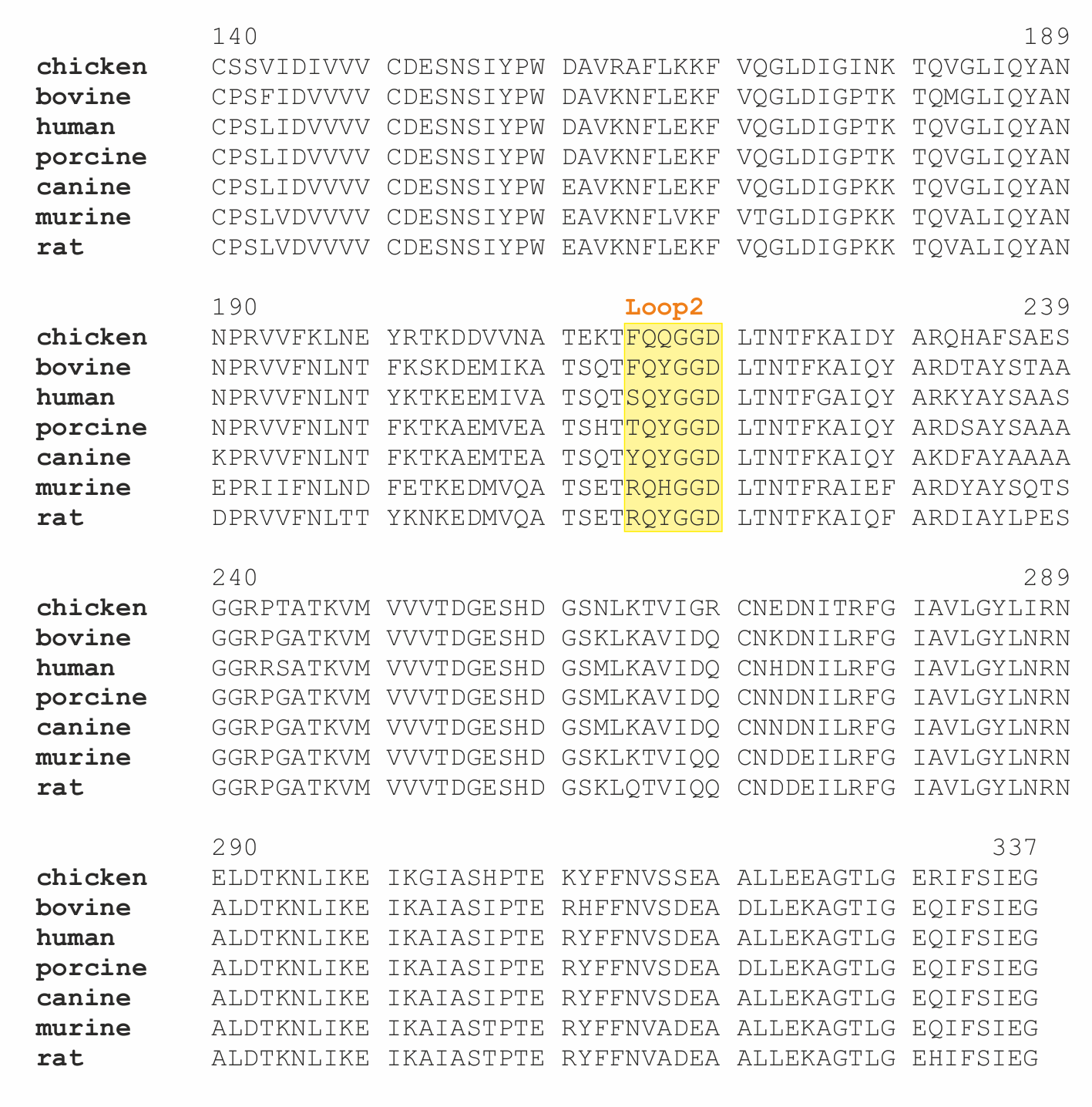

Supplement: S3 Fig — (A) Fragmentation scheme for the tryptic peptide, of the RCγ subunit containing the IIIG5 epitope. (B) NanoESI fragment ion spectrum of the RCγ peptide containing the IIIG5 epitope. It was obtained from a CID experiment on the ion mobility-separated doubly charged peptide precursor ions at m/z 942.40. The labelled peaks correspond to the fragment ions of this epitope peptide, as shown in (A). (TIF) [file pbio.2001492.s003.tif]

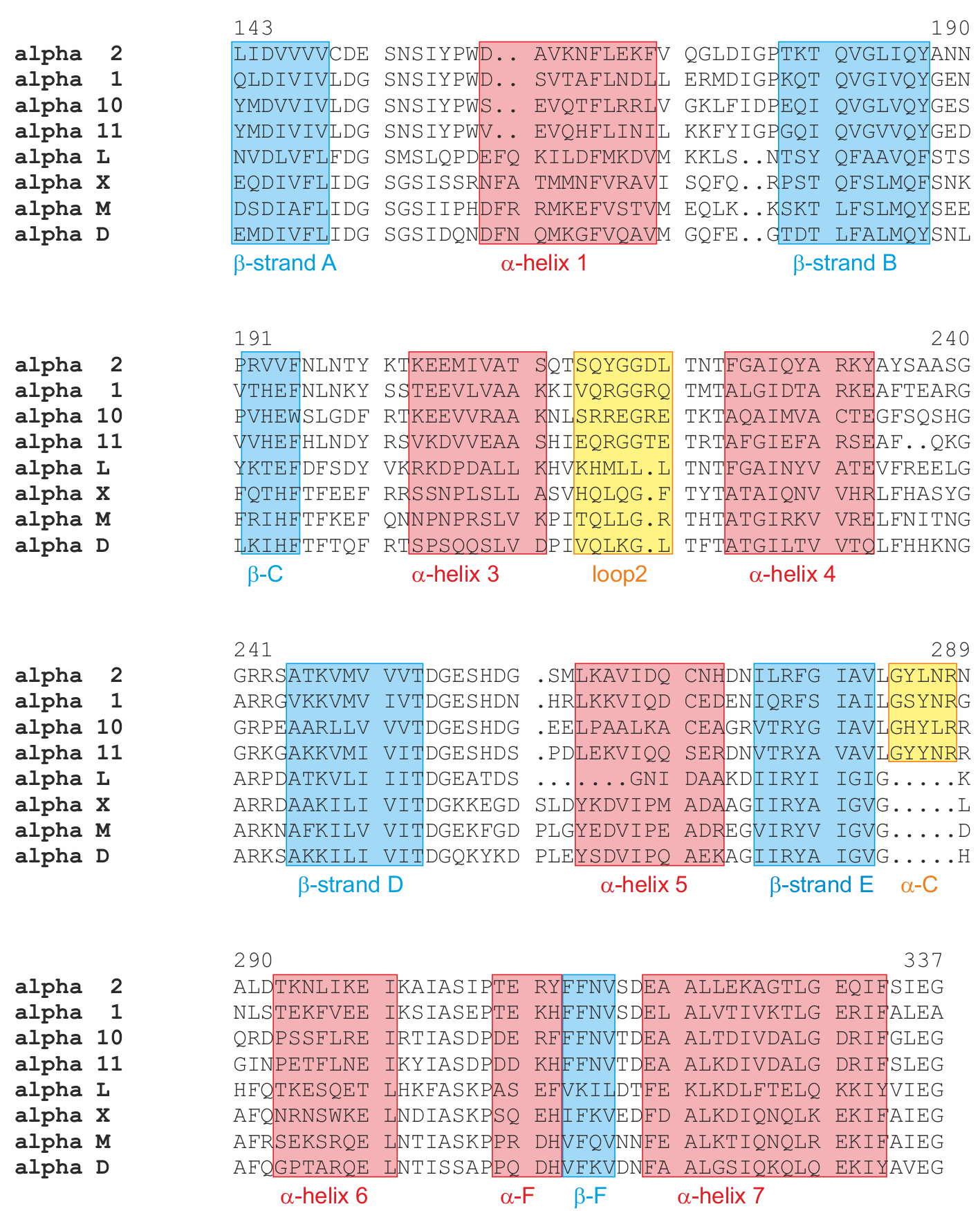

Supplement: S4 Fig — Sequence comparison of the integrin α2 A-domain from different vertebrate species. The loop 2 sequence S214QYGGD is highlighted in yellow and shows a high degree of homology between different species. Multiple sequence alignment was carried out with Clustal Omega Software from EMBL-EBI. (TIF) [file pbio.2001492.s004.tif]

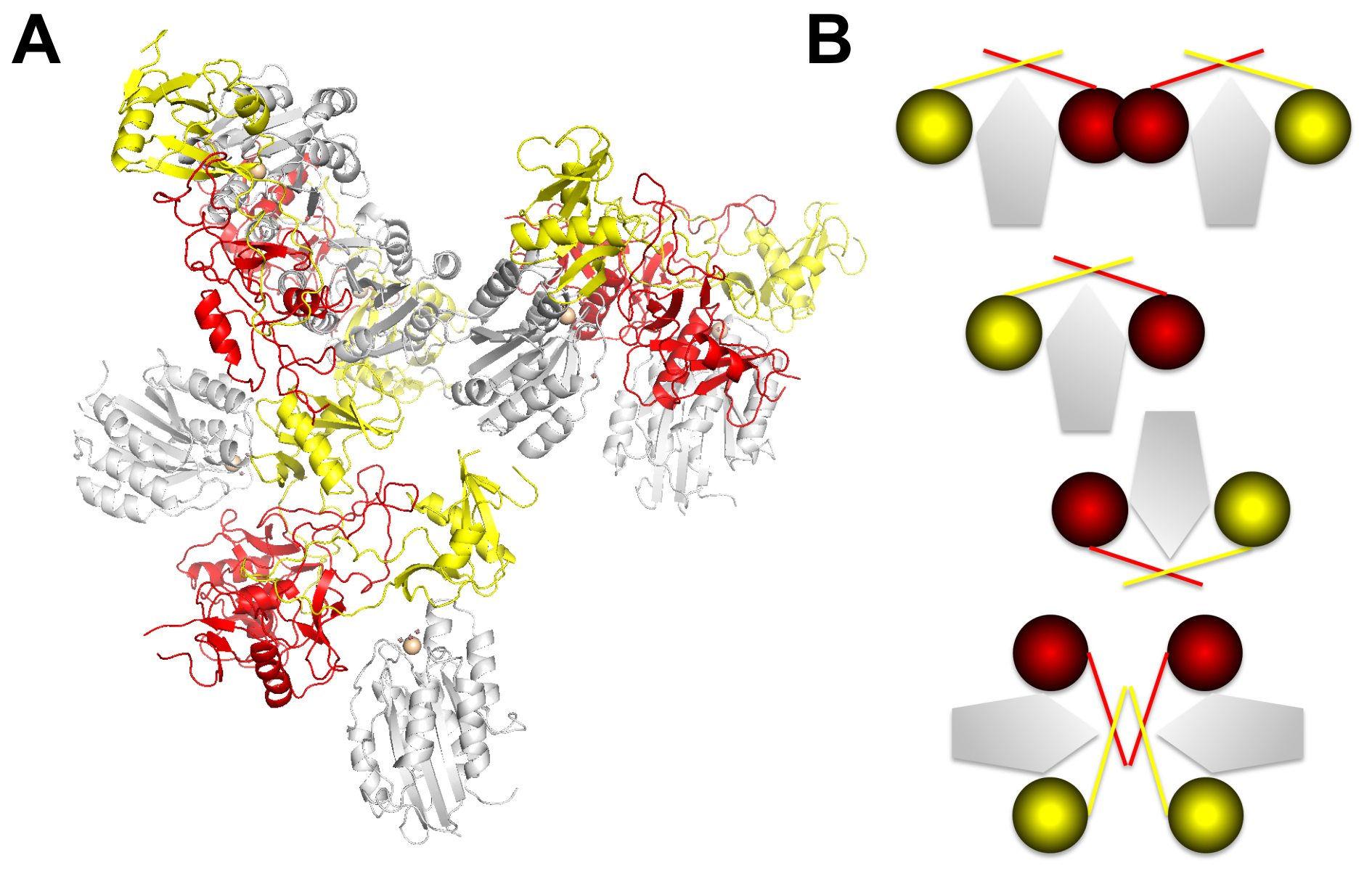

Supplement: S5 Fig — A comparison of A-domains from different human integrin α subunits. Integrin alpha subunits 1, 2, 10, and 11 belong to the subset of collagen binding integrins. They possess the characteristic helix C (yellow box, labelled α-C), which is absent in the A-domain of the leukocyte β2 integrins with their alpha subunits L, X, M, and D. Helix C of the integrin α2 subunit is the primary binding site for RCγδ and is only present in the “closed” conformation of its A domain. The secondary RC contact site of α2A is located within the loop 2 sequence S214QYGGD, (yellow box, labelled loop 2) and is specific to the integrin α2 chain. The secondary structure elements are indicated by the red (α-helices) and the blue (β-strands) boxes, respectively. The residue numbering refers to the integrin α2 sequence. Multiple sequence alignment was carried out with Clustal Omega Software from EMBL-EBI. (TIF) [file pbio.2001492.s005.tif]
